# Association Between Previous Stroke and Severe COVID-19: A Retrospective Cohort Study and an Overall Review of Meta-Analysis

**DOI:** 10.3389/fneur.2022.922936

**Published:** 2022-07-12

**Authors:** Huayao Huang, Junnian Chen, Shuangfang Fang, Xiaoling Chen, Xiaobin Pan, Hanhan Lei, Yixian Zhang, Hailong Lin, Qilin Yuan, Pincang Xia, Nan Liu, Houwei Du

**Affiliations:** ^1^Department of Rehabilitation, Fujian Medical University Union Hospital, Fuzhou, China; ^2^Department of Critical Care Medicine, Fujian Medical University Union Hospital, Fuzhou, China; ^3^Department of Neurology, Stroke Research Center, Fujian Medical University Union Hospital, Fuzhou, China; ^4^Department of Infectious Disease, Fujian Medical University Union Hospital, Fuzhou, China; ^5^Department of Critical Care Medicine, Fujian Provincial Hospital South Branch, Fuzhou, China; ^6^Department of Radiology, Fujian Medical University Union Hospital, Fuzhou, China; ^7^Fujian Center for Disease Control and Prevention, Fuzhou, China

**Keywords:** coronavirus disease 2019, cerebrovascular disease, stroke, outcome, overall review

## Abstract

**Objective:**

The objective of this study was to investigate the association between previous stroke and the risk of severe coronavirus disease 2019 (COVID-19).

**Methods:**

We included 164 (61.8 ± 13.6 years) patients with COVID-19 in a retrospective study. We evaluated the unadjusted and adjusted associations between previous stroke and severe COVID-19, using a Cox regression model. We conducted an overall review of systematic review and meta-analysis to investigate the relationship of previous stroke with the unfavorable COVID-19 outcomes.

**Results:**

The rate of severe COVID-19 in patients with previous stroke was 28.37 per 1,000 patient days (95% confidence interval [CI]: 10.65–75.59), compared to 3.94 per 1,000 patient days (95% CI: 2.66–5.82) in those without previous stroke (*p* < 0.001). Previous stroke was significantly associated with severe COVID-19 using a Cox regression model (unadjusted [hazard ratio, HR]: 6.98, 95% CI: 2.42–20.16, *p* < 0.001; adjusted HR [per additional 10 years]: 4.62, 95% CI: 1.52–14.04, *p* = 0.007). An overall review of systematic review and meta-analysis showed that previous stroke was significantly associated with severe COVID-19, mortality, need for intensive care unit admission, use of mechanical ventilation, and an unfavorable composite outcome.

**Conclusion:**

Previous stroke seems to influence the course of COVID-19 infection; such patients are at high risk of severe COVID-19 and might benefit from early hospital treatment measures and preventive strategies.

## Introduction

The coronavirus disease 2019 (COVID-19) due to acute respiratory syndrome coronavirus-2 (SARS-CoV-2) infection was first reported in December 2019 in Wuhan, China (1). COVID-19 became an unprecedented worldwide pandemic, affecting more than 500 million people (2). Cardiovascular risk factors, including ischemic heart disease, hypertension, and diabetes, are common in patients with COVID-19 infection and associated with mortality (3, 4). Notably, these results were based on observational studies. Recently, several studies used the Mendelian randomization design to show the causal associations between COVID-19 infection and cardiovascular risk factors, including hypertension, coronary heart disease, heart failure, and ischemic stroke (5–7). One previous study indicated that patients with severe COVID-19 were more likely to have the cerebrovascular disease (2.3 vs. 1.2%) (8). Stroke is a leading cause of complex acquired adult disability worldwide, causing impaired movement, cognition, and swallowing, which might expose stroke survivors to a high risk of severe pneumonia (9). Given the vast number of people living with previous stroke [80.1 million (74.1–86.3) prevalent cases worldwide in 2016] (10) and the unprecedented global spread of the COVID-19 pandemic, it is essential to know whether such individuals are at higher risk of severe COVID-19. We hypothesized that stroke survivors who had SARS-CoV-2 infection were more likely to experience severe COVID-19. Therefore, we investigated the association between previous stroke and severe COVID-19 in a retrospective study. The number of published systematic reviews regarding the relationship between previous stroke or cerebrovascular disease and COVID-19 prognosis continues to increase (11, 12). Therefore, it is urgent to conduct an overview to identify multiple relevant systematic reviews that address the same (or very similar) clinical questions and that include many (but not necessarily all) of the same primary studies (13). To the best of our knowledge, the overall review of systematic reviews addressing this topic is very limited. Therefore, we additionally performed an overall review of systematic reviews and meta-analyses on this topic. Our interest was to assess the methodological quality of the existing literature.

## Materials and Methods

### Study Design and Participants

The definition of the study cohort is patients with confirmed COVID-19. We performed a single-center, retrospective, observational study at Tumor Center of Union Hospital, Tongji Medical College, Huazhong University of Science and Technology (Wuhan, China). We conducted this study following the Strengthening the Reporting of Observational Studies in Epidemiology (STROBE) statement for observational studies (14). We analyzed consecutive patients admitted between 15 February and 14 March 2020 because of a COVID-19 based on World Health Organization interim guidance (15). We retrospectively reviewed the electronic medical records of these patients using a digital database. Laboratory confirmation of SARS-CoV-2 infection was performed by the local health authority as previously described (16). The exposure of interest was previous stroke, which was diagnosed based on the patient's medical record and history of prescription medication usage. The primary outcome was severe COVID-19. All clinical investigations were conducted according to the principles expressed in the Declaration of Helsinki. Written informed consent was waived due to the nature of our retrospective study of routine clinical data.

### Data Collection, Definitions of Exposure, and Outcome Measures

We retrospectively extracted the demographic, clinical, laboratory, and radiological data of 164 consecutive laboratory-confirmed COVID-19 patients with using a digital database, as described in our previous literature (17). Definitions of vascular risk factors (i.e., hypertension, diabetes, and dyslipidemia) were based on previous literature (18, 19). Obesity was defined as a body weight index >25 kg/m^2^ (18). We obtained and clarified data by direct communication with attending doctors and other healthcare providers if data were missing or uncertain from the medical records. Previous stroke was defined as chronic (prevalent) from 29 days after an incident stroke event, based on WHO criteria as rapidly developing clinical signs of disturbance of cerebral function lasting more than 24 h (15). Our primary outcome was severe COVID-19, defined as fever or suspected respiratory infection, with one of the following: respiratory rate > 30 breaths/min; severe respiratory distress; or SPO_2_ ≤ 93% on room air (15, 17). Our second outcome was mortality.

### Overall Review of Systematic Review and Meta-Analysis

Based on the *Cochrane Handbook for Systematic Reviews*, overviews of reviews of healthcare interventions integrate information from multiple similar systematic reviews to provide a single synthesis of relevant evidence for healthcare decision-making (20). To address the association between previous stroke and COVID-19 severity broader in scope and to understand the diversity present in the extant systematic review literature, we conducted an overall review of systematic reviews and meta-analysis.

### Literature Search and Screening

We performed an overall review of existing systematic review and meta-analysis on the association between previous stroke or cerebrovascular disease and COVID-19 outcomes (severe COVID-19, mortality, need for intensive care unit admission or mechanical ventilation, and an unfavorable composite outcome). We adhered to the preferred reporting items for systematic reviews and meta-analyses (PRISMA) guidelines (21, 22). We searched PubMed, Web of Science, and the Cochrane Database of Systematic Reviews to identify review-level relevant literature on the stroke and COVID-19 severity using the terms (“Coronavirus” OR “COVID-19” OR “SARS-CoV-2” OR “2019-nCoV”) AND (“stroke” OR “cerebrovascular disease” OR “cerebrovascular disorder” OR “infarction” OR “cerebral hemorrhage”), restricted to applications in humans from 2020 through to May 2022. We included studies evaluating the association between the preexisting stroke or cerebrovascular disease and COVID-19 severity and outcomes. We also manually screened out the relevant potential article in the references selected to obtain a comprehensive list of studies.

We included studies based on the following criteria: Cochrane systematic reviews and non-Cochrane systematic reviews and meta-analysis evaluating the association of preexisting stroke or cerebrovascular disease with the risk of severe COVID-19, mortality, need for intensive care unit (ICU) admission or mechanical ventilation, and an unfavorable composite outcome. The exclusion criteria were as follows: (1) pooled analyses not based on a systematic literature search; (2) systemic review without meta-analysis or those that did not provide evidence synthesis, narrative reviews, editorials, comments, and conference papers; (3) non-review articles including randomized controlled studies, cohort studies, case-control studies, case series, and case reports; (4) studies in newborns, infants, and children; and (5) insufficient or inaccurate data information provided. The exposure was preexisting stroke or cerebrovascular disease. We included those without stroke or cerebrovascular disease as a comparator. To avoid overlapping data from primary studies evaluated in more than one systematic review and meta-analysis, we followed the strategy developed by Pollock et al. (23). Briefly, this strategy is based on the following four questions: (1) Do Cochrane systematic reviews likely examine all relevant intervention comparisons and available data? (2) Do the Cochrane systematic reviews overlap? (3) Do the non-Cochrane systematic reviews overlap? and (4) Are researchers prepared and able to avoid double-counting outcome data from overlapping systematic reviews, by ensuring that each primary study's outcome data are extracted from overlapping systematic reviews only once? Two authors (H.H and S.F) blindly assessed the eligibility and bias risk and then extracted data. A consensus was reached by discussion when disagreements occurred.

### Methodological Quality of Included Systematic Reviews and Meta-Analyses

Two authors (S.W and H.L) used the methodological quality of systematic reviews (AMSTAR) to evaluate the quality of the studies included (24). We reported the data in a descriptive way since there are no clear recommendations on how to report the results of the AMSTAR quality appraisal tool (24).

### Statistical Analysis

For the cohort study, data were summarized with mean value with standard deviations or median value with interquartile range, and were categorized as counts with percentages. *T*-test or Mann–Whitney test was used to compare the differences in continuous variables, and the chi-square test or Fisher's exact test to compare the differences in categorical variables where appropriate. The absolute event rate per 1,000 patient days for severe COVID-19 and death during our observation period were calculated. The Kaplan–Meier probabilities of severe COVID-19 and mortality during our observation period stratified by previous stroke were calculated. Univariable and multivariable Cox regression analyses were used to evaluate the association between previous stroke (exposure) and severe COVID-19 (outcome). Variables with *p* < 0.1 in the univariable analysis were included in the age- and sex-adjusted model. We further included cardiovascular risk factors (hypertension, diabetes, and obesity) in addition to age and sex in the multivariable regression model. All statistical analyses were performed using SPSS for Windows (SPSS 25.0, IBM, Inc., Chicago, IL, USA).

## Results

Among 164 consecutive COVID-19 patients, six (3.7%) patients had previous stroke. Table 1 shows the demographics, clinical, and radiological characteristics between patients with and without previous stroke.

**Table 1:** Characteristics at baseline in COVID-19 patients with and without previous stroke history.

**Variables**	**Total**	**With stroke**	**Without stroke**	***P***
	**(*n* = 164)**	**(*n* = 6)**	**(*n* = 158)**	
Age, (y) mean ± SD	61.8 ± 13.6	74.5 ± 9.2	61.3 ± 13.5	0.02
Male, *n* (%)	84 (51.2)	3(50.0)	81(51.3)	>0.99
Current smoker, *n* (%)	17 (10.4)	1(16.7)	16 (10.1)	>0.99
Often drinker, *n* (%)	3 (1.8)	0	3 (1.9)	>0.99
Hypertension, *n* (%)	52 (31.7)	5 (83.3)	47 (29.7)	0.02
Diabetes, *n* (%)	31 (18.9)	1(16.7)	30 (19.0)	>0.99
COPD, *n* (%)	12 (7.3)	2 (33.3)	10 (6.3)	0.09
CHD, *n* (%)	21 (12.8)	2 (33.3)	19 (12.0)	0.36
Obesity, *n* (%)	55 (33.5)	3 (50.0)	52 (32.9)	0.67
Digestive disease, *n* (%)	15 (9.1)	1(16.7)	14 (8.9)	>0.99
Tumor, *n* (%)	13 (7.9)	1 (16.7)	12 (7.6)	0.97
Immunosuppresive, *n* (%)	3 (1.8)	0	3 (1.9)	>0.99
Wet market exposure, *n* (%)	2 (1.2)	0	2 (1.3)	>0.99
**Clinical manifestion**				
Fever, *n* (%)	115 (70.1)	4 (66.7)	111 (70.3)	>0.99
Dry cough, n, (%)	104 (63.4)	4 (66.7)	100 (63.3)	>0.99
Productive cough, *n* (%)	23 (14.0)	0	23 (14.6)	0.60
Fatigue, *n* (%)	57 (34.8)	2 (33.3)	55 (34.8)	>0.99
Musle or joint ache, *n* (%)	21(12.8)	0	21 (13.3)	>0.99
Thoracalgia, *n* (%)	31(18.9)	0	31(19.6)	0.60
Sore throat, *n* (%)	23 (14.0)	1 (16.7)	22 (13.9)	>0.99
Diarrhea, *n* (%)	13 (7.9)	1 (16.7)	12 (7.6)	0.97
Catarrh, *n* (%)	6 (3.7)	0	6 (3.8)	>0.99
Anorexia, *n* (%)	48 (29.3)	3 (50.0)	45 (28.5)	0.50
Short of breath, *n* (%)	65 (39.6)	2 (33.3)	63 (39.9)	>0.99
Headache	19 (11.6)	3 (50.0)	16 (10.1)	0.02
Total symptoms, IQR	3 (2–4)	4 (2–5)	3 (2–4)	0.68
**Regular blood test**				
Decreased leucocytes, *n* (%)	11 (6.7)	1 (16.7)	10 (6.3)	0.87
Decreased lymphocytes, *n* (%)	55 (33.5)	4 (66.7)	51 (32.3)	0.19
Decreased hemoglobin, *n* (%)	42 (25.6)	3 (50.0)	39 (24.7)	0.36
Decreased platelets, *n* (%)	14 (8.5)	2 (33.3)	12 (7.6)	0.14
**CT findings**, ***n*** **(%)**				
Unilateral pneumonia, *n* (%)	26 (15.9)	1 (16.7)	25 (15.8)	0.58
Bilateral pneumonia, *n* (%)	86 (52.4)	2 (33.3)	84 (53.2)	
Multiple mottling and Ground-glass opacity, *n* (%)	52 (31.7)	3 (50.0)	49 (31.0)	

*COVID-19, coronavirus disease 2019; COPD, chronic obstructive pulmonary disease; CHD, chronic heart disease; CT, computed tomography; IQR, interquartile range*.

*Decreased means below the lower limit of the normal range. Leucocytes (× 10^9^ per L; normal range 3.5–9.5); lymphocytes (× 10^9^ per L; normal range 1.1–3.2); platelets (× 10^9^ per L; normal range 125.0–350.0); hemoglobin (g/L; normal range 130.0–175.0)*.

**Figure 1:**
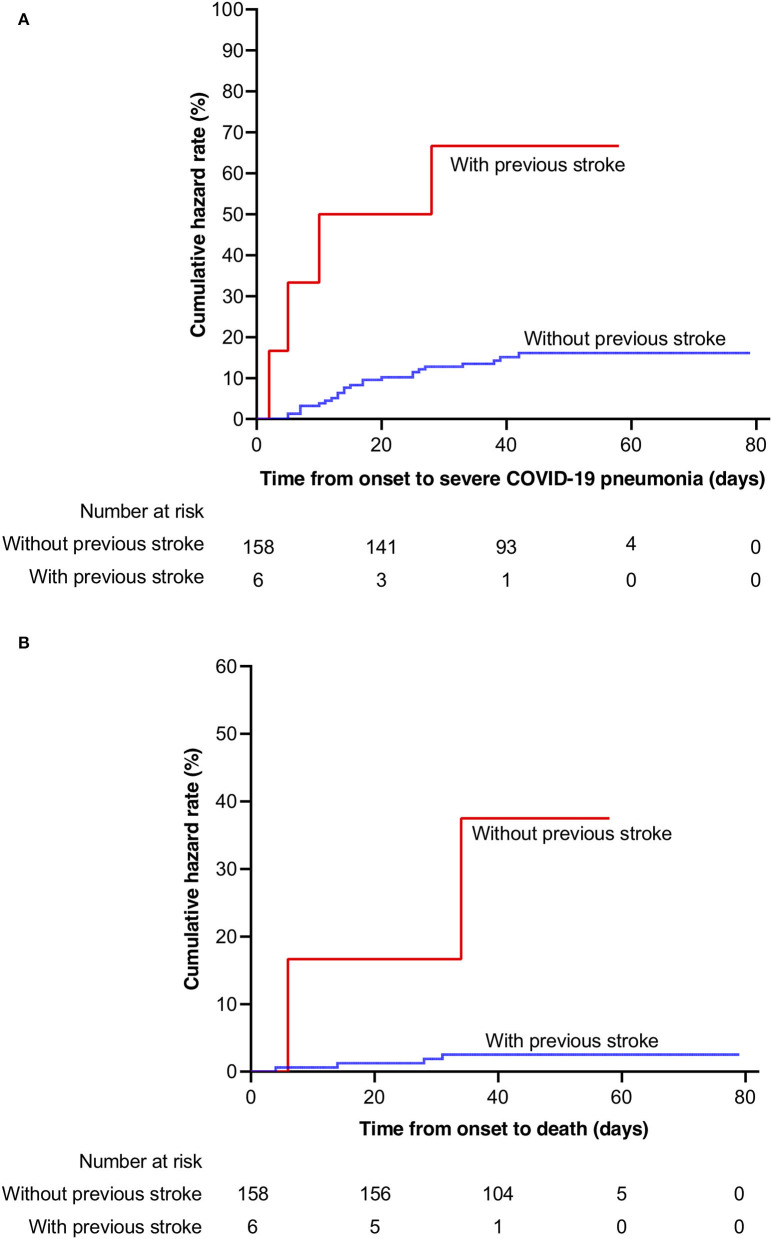
Kaplan–Meier probabilities in patients with and without previous stroke. **(A)** severe COVID-19; **(B)** mortality. COVID-19, coronavirus disease 2019.

The severe COVID-19 event rate in patients with previous stroke was 28.37 per 1,000 patient days (95% CI: 10.65–75.59), compared to 3.94 per 1,000 patient days (95% CI: 2.66–5.82) in those without previous stroke. The death event rate in patients with previous stroke was 9.66 per 1,000 patient days (95% CI: 2.42–38.63) compared to 0.58 per 1,000 patient days (95% CI: 0.22–1.54) in those without previous stroke. In Kaplan–Meier analysis, severe COVID-19 event and death were more frequent in patients with previous stroke compared with those without stroke (Log-rank test, *p* < 0.001, respectively, Figure 1). In univariable Cox regression analysis, patients with previous stroke were at a higher risk of having severe COVID-19 (HR [hazard ratio]: 6.98, 95% CI: 2.42–20.16, *p* < 0.001). After adjustment for age (per 10 years increasing) and sex, previous stroke remained significantly associated with severe COVID-19 (adjusted HR: 4.62, 95% CI: 1.52–14.04, *p* = 0.007, Table 2). This association remained after additional adjustment for hypertension, diabetes, and obesity (adjusted HR: 4.66, 95% CI: 1.54–14.12, *p* = 0.006).

**Table 2:** Association between previous stroke and severe COVID-19 pneumonia.

	**Unadjusted**		**Age- and sex-adjusted**	
	**HR (95% CI)**	***p*-value**	**HR (95% CI)**	***p*-value**
Age (per 10 years)	1.87 [1.39–2.51]	<0.001		
Male sex	2.34 [1.06–5.14]	0.04		
Hypertension	1.91 [0.92–3.98]	0.08	1.07 [0.48–2.40]	0.87
Previous stroke	6.98 [2.42–20.16]	<0.001	4.62 [1.52–14.04]	0.007
Obesity	2.03 [0.98–4.21]	0.06	1.62 [0.77–3.43]	0.21
CT findings	Ref	0.05	Ref	0.67
Unilateral pneumonia	1.12 [0.31–4.01]		1.15 [0.32–4.14]	
Bilateral pneumonia	2.75 [0.79–9.48]		1.60 [0.44–5.73]	
**Multiple mottling and ground-glass opacity**				
Decreased lymphocytes	4.46 [2.07–9. 60]	<0.001	3.30 [1.50–7.25]	0.003
Decreased leucocytes	3.51 [1.34–9.23]	0.011	2.14 [0.74–6.16]	0.16
Decreased hemoglobin	3.63 [1.75–7.52]	0.001	3.37 [1.55–7.33]	0.002
Decreased platelets	4.69 [2.00–11.01]	<0.001	2.95 [1.22–7.11]	0.02

*COVID-19, coronavirus disease 2019; CT, computed tomography; HR, hazard ratio*.

*Decreased means below the lower limit of the normal range*.

Detailed information of six patients with previous ischemic stroke is shown in Table 3. Of six patients, four (66.7%) were dependent on their caregivers at baseline, indicating a population with a severe burden of baseline disability, two (33.3%) experienced a new stroke event, and two (33.3%) died during hospitalization. Five (83.3%) patients had D-dimer > 0.5 μg/ml.

**Table 3:** Characteristics and outcome of patients with previous stroke (*n* = 6).

**Variables**	**Patient 1**	**Patient 2**	**Patient 3**	**Patient 4**	**Patient 5**	**Patient 6**
Sex	Male	Male	Female	Female	Female	Male
Age, (y)	78	74	61	86	81	67
Stroke subtypes	Cardiac	LAA	SAO	LAA	Undetermined	LAA
Baseline mRS score	5	4	2	4	2	3
COPD	No	No	No	No	Yes	Yes
Hypertension	Yes	No	Yes	Yes	Yes	Yes
Diabetes	No	No	No	No	Yes	No
CHD	Yes	No	No	Yes	No	No
Decreased lymphocytes	Yes	No	No	No	Yes	No
Decreased hemoglobin	Yes	No	Yes	No	Yes	No
Albumin <30 g/L	Yes	Yes	No	No	Yes	Yes
ALT > 40I U/L	Yes	No	Yes	No	No	Yes
AST > 40I U/L	Yes	Yes	No	Yes	No	Yes
D-dimer (mg/L)	0.23	8.16	2.96	1.11	0.71	1.23
Chest CT Findings	2	3	2	3	1	3
Severe COVID-19	Yes	Yes	No	No	Yes	Yes
Outcomes at discharge	Death	Death	Cured	Not cured	Cured	Cured
Recurrent stroke	Yes	Yes	No	No	No	No
Previous stroke prior to onset of COVID-19	72 d	> 4 y	> 1 y	> 12 y	> 1 y	>10 m

*COPD, chronic obstructive pulmonary disease; CHD, chronic heart disease; ALT, serum alanine aminotransferase; AST, serum aspartate aminotransferase; CT, computed tomography; CT findings: 1, unilateral pneumonia, 2, bilateral pneumonia, 3, multiple mottling and ground-glass opacity; LAA, large artery atherosclerosis; SAO, small artery occlusion; stroke subtype is based on TOAST (Trial of Org 10172 in Acute Stroke Treatment) criteria*.

### Overall Review of Systemic Review and Meta-Analysis

**Figure 2:**
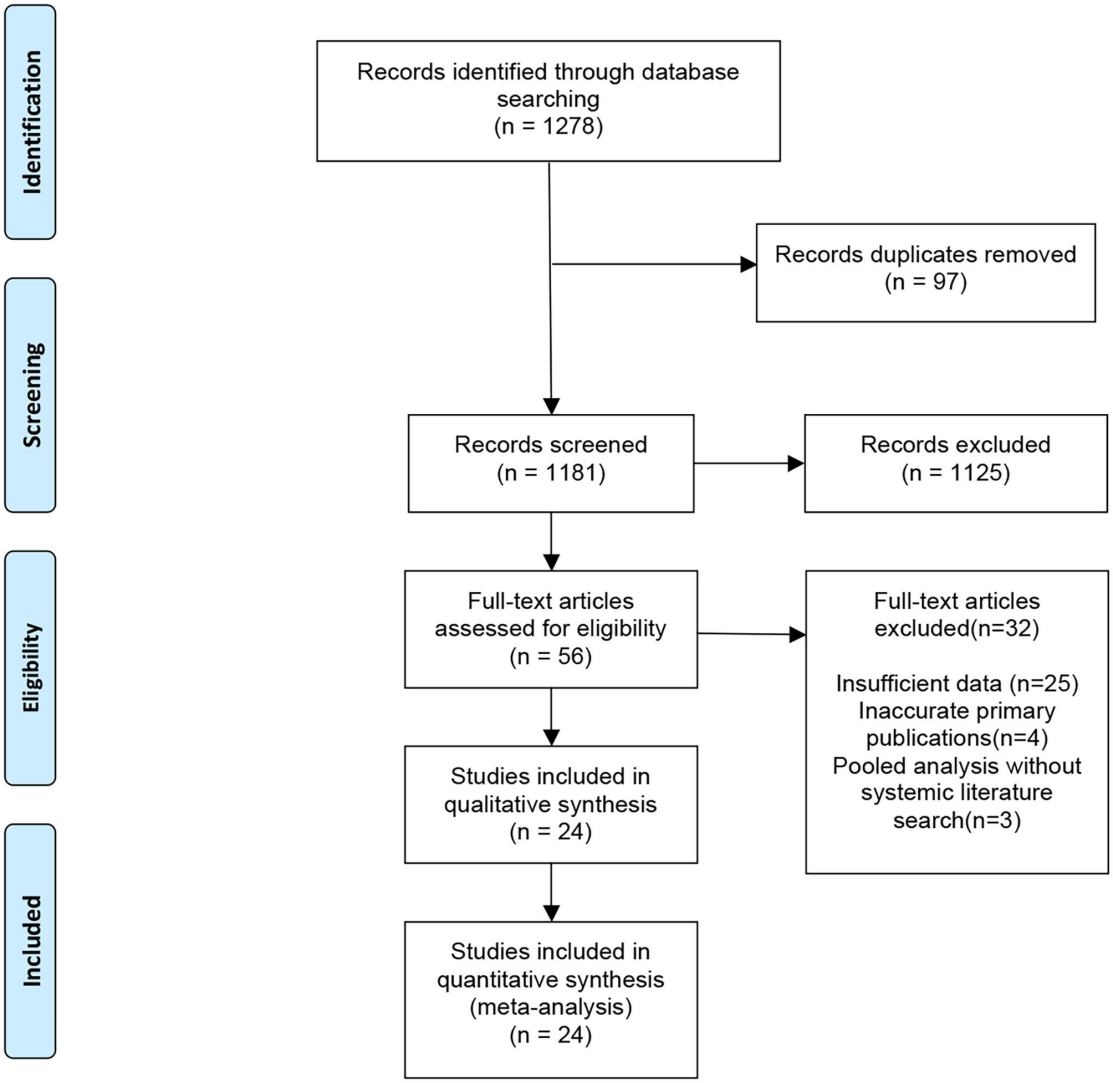
Flowchart of literature selection.

An initial search identified a total of 1,278 potentially relevant meta-analyses. After the exclusion of 97 duplicate articles, we excluded 1,181 studies after screening the title and abstract, followed by excluding 32 publications after full-text screening. A total of 24 published meta-analyses (25–48) met our inclusion criteria (Figure 2). Most meta-analyses were conducted in 2020, with only three in 2021. Among 172 primary publications, 106 were conducted in China. Essential baseline characteristics of the included studies are listed in Table 4. The summary of findings in the included meta-analyses is shown in Table 5. Quality appraisal using the AMSTAR method of included systematic reviews and meta-analyses is shown in Table 6.

**Table 4:** Characteristics of included systematic review and meta-analyses.

**References**	**Last search date**	**Included studies**	**Exposure**	**Severe COVID-19**	**Mortality**	**ICU**	**Mechanical Ventilation**	**Composite endpoint**	**Database**
Barek et al. (25)	23 May 2020	15	Cerebrovascular disease	✓	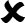	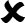	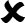	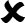	PubMed, ScienceDirect and BMC Journal database
Del Sole et al. (26)	28 May 2020	5	Cerebrovascular disease	✓	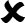	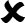	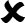	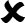	PubMed, ISI Web of Science, SCOPUS and Cochrane
Fang et al. (27)	5 April 2020	14	Cerebrovascular disease	✓	✓	✓	✓	✓	Pubmed, medRxiv or bioRxiv
Fernández et al. (28)	28 May 2020	16	Cerebrovascular disease	✓	✓	✓	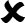	✓	MEDLINE, bioRXiv, and MedRXiv
Figliozzi et al. (29)	24 April 2020	17	Cerebrovascular disease	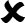	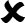	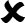	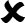	✓	PubMed/MEDLINE and Scopus
Florez-Perdomo et al. (30)	May 2020	7	Cerebrovascular disease	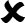	✓	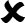	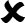	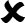	PubMed/Embase/EBSCO Host/Scopus/Science Direct/Medline/LILACS
Gao et al. (31)	10 October 2020	37	Cerebrovascular disease/stroke	✓	✓	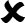	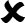	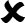	PubMed, EMBASE.com, Web of Science and Cochrane
Katzenschlager et al. (32)	31 May 2020	18	Cerebrovascular disease	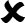	✓	✓	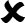	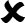	Medline [PubMed] and Web of Science Core Collection (bioRxiv and medRxiv)
Li et al. (33)	14 April 2020	4	Cerebrovascular disease	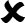	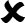	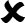	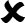	✓	PubMed, Embase, and Cochrane Library data- bases
Li at al. (34)	22 November 2021	47	Stroke	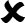	✓	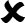	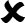	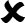	PubMed, Web of Science, Scopus, and Embase
Li et al. (35)	February 2021	14	Cerebrovascular disease	✓	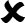	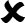	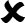	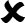	PubMed, Embase, Web of science and Cochrane Library
Patel et al. (36)	April 30 2020	9	Cerebrovascular disease	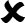	✓	✓	✓	✓	PubMed, Web of Science, and Sco- pus
Pranata et al. (37)	10 April 2020	12	Cerebrovascular disease	✓	✓	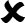	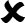	✓	PubMed, SCOPUS, EuropePMC, and Cochrane Central Database
Ramphul et al. (38)	15 October 2020	25	Cerebrovascular disease	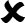	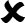	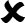	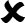	✓	Medline, Scopus, Wangfang, Web of Science, Research Square, medrxiv, and Google Scholar
Siepmann et al. (39)	15 April 2020	12	Cerebrovascular disease	✓	✓	✓	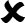	✓	PubMed, EMBASE, Cochrane Library databases
Singh et al. (40)	April 23, 2020	7	Cerebrovascular disease	✓	✓	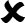	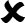	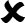	Medline, Scopus and the World Health Organization website
Ssentongo et al. (41)	9 July 2020	25	Cerebrovascular disease	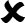	✓	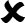	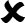	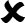	MEDLINE, SCOPUS, OVID, Cochrane, medrxiv.
Wang et al. (42)	1 March 2020.	3	Cerebrovascular disease	✓	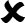	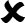	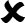	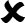	PubMed, Cochrane Library, Embase, and other databases
Wang et al. (43)	30 April 2020	6	Cerebrovascular disease	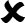	✓	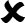	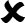	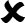	PubMed, Web of Science, and China National Knowledge Infrastructure (CNKI)
Xu et al. (44)	10 August2020	12	Cerebrovascular disease	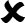	✓	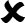	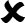	✓	PubMed, Web of Science, EMBASE
Yin et al. (45)	18 January 2021	41	Cerebrovascular disease	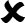	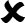	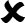	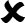	✓	PubMed, Web of Science, CNKI
Yu et al. (46)	25 July 2020	31	Stroke	✓	✓	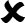	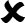	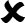	PubMed, Wed of Science, CNKI, MedRxiv and BioRxiv
Zhang et al. (47)	29 September 2020	11	Cerebrovascular disease	✓	✓	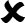	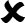	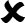	PubMed, EMBASE, and MEDLINE
Zhou et al. (48)	25April 2020	8	Cerebrovascular disease	✓	✓	✓	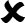	✓	PubMed, Embase,Cochrane

*COVID-19, coronavirus disease 2019; ICU, intensive care unit*.

*“✓” represents data provided; “
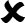
” represents data not provided*.

**Table 5:** Summary of study findings.

**Reference**	**Effect model**	**Effect measure**	**Severe COVID-19**	**Mortality**	**ICU**	**Mechaniacl ventilation**	**Composite endpoint**
Barek et al. (25)	RE	OR	3.78 [2.22–6.43]	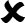	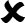	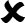	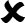
Del Sole et al. (26)	RE or FE	OR	3.66 [1.73–7.72]	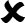	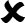	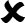	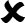
Fang et al. (27)	RE	RR	2.77 [1.70–4.52]	4.55 [2.60–7.94]	4.52 [2.48–8.25]	4.03 [1.72- 9.02]	4.08 [2.03–8.17]
Fernández et al. (28)	RE	RR	2.25 [1.51–3.35]	2.65 [1.73–4.06]	1.88 [0.89–3.97]	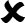	4.59 [1.92–10.98]
Figliozzi et al. (29)	RE	OR	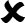	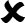	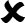	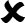	2.93 [1.64–5.24]
Florez-Perdomo et al. (30)	RE	OR	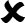	2.78 [1.42–5.46]	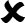	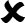	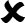
Gao et al. (31)	RE	OR [cerebrovascular]	3.10 [2.21–4.36]	3.45 [2.46–4.84]	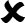	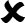	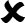
Gao et al. (31)	RE	OR [stroke]	1.95 [1.11–3.42]	1.79 [0.76–4.23]	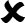	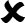	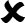
Katzenschlager et al. (32) []	RE	OR	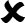	3.45 [2.42–4.91]	5.88 [2.35–14.73]	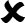	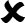
Li et al. (33)	FE	OR	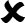	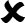	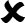	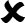	2.68 [1.29–5.57]
Li at al. (34)	RE	Pooled effect	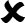	1.30 [1.16–1.44]	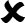	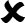	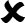
Li et al. (35)	RE	OR	2.47 [1.54–3.97]	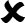	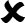	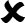	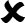
Patel et al. (36)	RE	OR	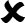	1.42 [1.14–1.77]	1.82 [1.25–2.69]	1.33 [1.09–1.63]	2.67 [1.75–4.06]
Pranata et al. (37)	RE	RR	1.88 [1.00–3.51]	2.38 [1.92–2.96]	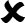	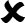	2.04 [1.43–2.91]
Ramphul et al. (38)	RE or FE	OR	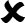	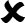	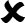	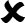	2.63 [1.80–3.85]
Siepmann et al. (39)	RE	RR	1.44 [1.22–1.71]	2.18 [1.75–2.7]	2.79 [1.83–4.24]	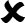	2.07 [1.52–2.87]
Singh et al. (40)	RE	RR	1.73 [0.74–4.05]	2.48 [2.14–2.86]	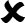	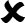	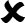
Ssentongo et al. (41)	RE	OR	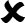	2.16 [0.97–4.80]	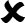	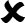	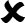
Wang et al. (42)	FE	OR	3.89 [1.64–9.22]	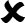	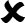	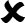	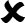
Wang et al. (43)	RE or FE	RR	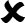	4.78 [3.24–7.03]	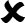	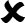	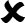
Xu et al. (44)	RE	Pooled effect	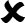	1.78 [1.04–3.07]	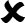	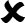	2.05 [1.34–3.16]
Yin et al. (45)	RE	OR	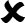	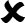	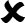	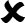	3.70 [2.51–5.45]
Yu et al. (46)	RE or FE	OR	3.004 [2.097–4.303]	23.477 [3.050–180.735]	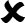	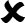	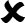
Zhang et al. (47)	FE	OR	4.45 [2.94–6.74]	6.47 [3.55–11.78]	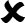	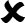	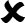
Zhou et al. (48)	RE	OR	2.24 [1.26–3.98]	13.27[0.71–249.04]	20.20 [2.34–174.44]	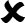	2.74 [1.59–4.74]

*COVID-19, coronavirus disease 2019; ICU, intensive care unit; RE, random-effects; FE, fixed-effects; OR, odds ratio; RR, risk ratio*.

*“
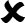
” represents data not provided*.

**Table 6:** Quality appraisal of included systematic reviews.

**References**	**1. Was an ‘a priori' design provided?**	**2. Was there duplicate study selection and data extraction?**	**3. Was a comprehensive literature search performed?**	**4. Was the status of publication (i.e., gray literature) used as an inclusion criterion?**	**5. Was a list of studies (included and excluded) provided?**	**6. Were the characteristics of the included studies provided?**	**7. Was the scientific quality of the included studies assessed and documented?**	**8. Was the scientific quality of the included studies used appropriately in formulating conclusions?**	**9. Were the methods used to combine the findings of studies appropriate?**	**10. Was the likelihood of publication bias assessed?**	**11. Was the conflict of interest stated?**
Barek et al. (25)	No	Yes	Yes	No	Yes	Yes	Yes	Yes	Yes	Yes	Yes
Del Sole et al. (26)	No	Yes	No	No	No	Yes	No	Yes	Yes	No	Yes
Fang et al. (27)	No	Yes	Yes	Yes	Yes	Yes	Yes	Yes	Yes	Yes	Yes
Fernández et al. (28)	Yes	Yes	No	Yes	Yes	Yes	Yes	Yes	Yes	Yes	Yes
Figliozzi et al. (29)	Yes	Yes	Yes	No	Yes	Yes	Yes	Yes	Yes	Yes	Yes
Florez-Perdomo et al. (30)	No	Yes	Yes	No	No	Yes	Yes	Yes	Yes	Yes	Yes
Gao et al. (31)	Yes	Yes	Yes	No	No	Yes	Yes	Yes	Yes	Yes	Yes
Katzenschlager et al. (32)	Yes	Yes	Yes	Yes	No	Yes	Yes	Yes	Yes	Yes	Yes
Li et al. (33)	No	Yes	Yes	No	No	Yes	Yes	Yes	Yes	No	Yes
Li at al (34)	No	Yes	Yes	Yes	Yes	Yes	Yes	Yes	Yes	Yes	Yes
Li et al. (35)	No	Yes	Yes	No	No	Yes	Yes	Yes	Yes	Yes	Yes
Patel et al. (36)	No	Yes	Yes	No	No	Yes	Yes	Yes	Yes	No	Yes
Pranata et al. (37)	No	Yes	Yes	No	No	Yes	No	Yes	Yes	No	Yes
Ramphul et al. (38)	No	Yes	Yes	Yes	No	Yes	No	Yes	Yes	No	Yes
Siepmann et al. (39)	No	Yes	Yes	No	No	Yes	No	Yes	Yes	Yes	Yes
Singh et al. (40)	Yes	Yes	Yes	Yes	No	Yes	No	Yes	Yes	Yes	Yes
Ssentongo et al. (41)	Yes	Yes	Yes	Yes	No	Yes	Yes	Yes	Yes	Yes	Yes
Wang et al. (42)	No	Yes	Yes	No	No	Yes	No	Yes	Yes	Yes	Yes
Wang et al. (43)	No	Yes	Yes	No	No	Yes	No	Yes	Yes	Yes	Yes
Xu et al. (44)	No	Yes	Yes	No	No	Yes	Yes	Yes	Yes	Yes	Yes
Yin et al. (45)	Yes	Yes	Yes	No	No	Yes	No	Yes	Yes	Yes	Yes
Yu et al. (46)	No	Yes	Yes	Yes	No	Yes	Yes	Yes	Yes	Yes	Yes
Zhang et al. (47)	Yes	Yes	Yes	No	No	Yes	Yes	Yes	Yes	Yes	Yes
Zhou et al. (48)	Yes	Yes	Yes	No	Yes	Yes	No	Yes	Yes	Yes	Yes

### Description of Studies: Strength and Balance

We calculated the corrected covered area (CCA) using the method by Pieper et al. as a measure of overlap by dividing the frequency of repeated occurrences of the index publication in other reviews by the product of index publications and reviews, reduced by the number of index publications. CCA = (*N* – *r*) / (*c* × *r* – *r*), where *N* is the number of included publications (including repeated counting) in evidence synthesis, *r* is the number of rows (number of index publications), and *c* is the number of columns (meta-analyses) (49). The 24 meta-analyses had included unrepeated 172 primary publications, and the CCA was 3.97% [(329–172)/(24 × 172–172)], representing a low overlap of primary publications (Supplementary Table S1).

### Association Between Preexisting Cerebrovascular Disease and Severe COVID-19

A total of 13 meta-analyses reported the association between preexisting cerebrovascular disease and severe COVID-19. Two studies reported the effect of previous stroke on severe COVID-19 (34, 46). One study (31) reported both cerebrovascular disease and stroke as exposures. All the meta-analyses showed an association between previous stroke (cerebrovascular disease) and higher risk of severe COVID-19 except one study (RR: 1.73, 95% CI: 0.74–4.0) (40). Table 5 shows that the effect estimates were highly consistent with a minimum OR of 1.44 (95% CI: 1.22–1.71) (39) and a maximum OR of 4.45 (95% CI: 2.94–6.74) (47).

### Association Between Preexisting Cerebrovascular Disease and Mortality

A total of 16 meta-analyzes provided the pooled effect estimates on the risk of mortality. There was a heterogeneity for this outcome, but the pooled effect estimates consistently support an association between preexisting cerebrovascular disease and mortality across all 16 studies and were statistically significant in 14 studies (Table 5).

### Association Between Preexisting Cerebrovascular Disease and ICU Admission

Among six systematic review and meta-analyses that provided data on the risk of ICU admission, five showed that preexisting stroke was associated with a higher risk of ICU admission. This association was lost in only one study (28) (RR: 1.88, 95% CI: 0.89–3.97, Table 5).

### Association Between Preexisting Cerebrovascular Disease and the Need for Mechanical Ventilation

Two meta-analyses (27, 36) provided data regarding the association between preexisting cerebrovascular disease and the need for mechanical ventilation (OR: 4.03, 95% CI: 1.72–9.02, and OR: 1.33, 95% CI: 1.09–1.63, respectively, Table 5).

### Association Between Preexisting Cerebrovascular Disease and an Unfavorable Composite Outcome

A total of 11 systematic review and meta-analyses provided data on the risk of an unfavorable composite COVID-19, consistently showing that preexisting cerebrovascular disease was associated with a higher risk of developing an unfavorable composite outcome (Table 5).

### Quality Appraisal of Included Meta-Analyses

Table 6 displays the results of each item of the AMSTAR tool for each meta-analysis, where positive answers are related to a low risk of bias. Less than half were prospectively registered, and no meta-analysis met all quality criteria, suggesting a moderate quality of evidence for most meta-analyses (50).

## Discussion

Our observational study showed that patients with previous stroke were more likely to experience severe COVID-19 and mortality compared to those without previous stroke. This observation was consistent with our overall review of meta-analysis that provided an update on the impact of previous cerebrovascular disease on the COVID-19 prognosis. These findings suggest that this population needs timely medical treatment at hospital admission as well as a preventive strategy (i.e., priority COVID-19 vaccination).

Since COVID-19 is a new disease but may exist for a very long time, early identification of endangered individuals may be helpful to plan more appropriate preventive therapy and eventually improve survival and optimize the allocation of healthcare resources. The present comprehensive overall review of meta-analysis collected the most recent synthetic evidence to show the effect of previous cerebrovascular disease on the prognosis of the SARS-CoV-2 infection. We used an evidence-based decision tool to make transparent and informed inclusion decisions in our overviews, reducing potential sources of bias that might skew pooled effect estimates. The qualitative implications applied to different data sources with a broad geographic distribution allow a wide generalizability. Therefore, it is safe to conclude that patients with previous stroke were at a higher risk of experiencing severe COVID-19 and mortality. Given the limited medical and financial resources, knowing the exact magnitude of risk is essential for better protection and identification of the most vulnerable individuals.

Infectious disease remains one major threat to stroke survivors. Hospital and community-acquired infections are associated with morbidity, mortality, and impaired functional outcome in stroke patients (51). Stroke-related deficits (i.e., dysphagia, motor dysfunction, and cognitive impairment) are associated with a higher risk of infection (52). During the early COVID-19 pandemic, some stroke survivors with poor neurological function may have difficulty in seeking help from caregivers. Of six, four (66.7%) stroke patients in our cohort had poor neurological function defined as a modified Rankin Scale >2 points and had limited access in getting help from their caregivers, which might be a contributor to their severe COVID-19.

The impact of previous stroke or cerebrovascular disease on the unfavorable COVID-19 outcomes may be explained by several factors. First, stroke patients were older and more often likely to have hypertension and diabetes, sharing dysregulated innate immune response that might influence severe COVID-19 infection (53). Second, an elevated D-dimer level might be an indicator of an unfavorable outcome of COVID-19. We found that 15 (88.2%) of 17 patients with severe COVID had elevated D-dimer compared to 41 (46.1%) of 89 in those without severe COVID (data available in 106 patients). Moreover, five (83.3%) of six patients with previous stroke had a higher serum level of D-dimer, which might have contributed to their risk of severe COVID-19. Our finding was supported by a meta-analysis of 17,052 patients that showed that serum D-dimer level was significantly higher in patients with severe COVID-19 (weighted mean difference: 0.60, 95% CI: 0.49–0.71) (54). An elevated D-dimer level has emerged as a biomarker of COVID-19 severity and associated coagulopathy, suggesting the potential impact of coagulopathy on COVID-19 prognosis (55). Third, hypoproteinemia was found in four (66.7%) of six patients who had previous stroke, which might indicate a high prevalence of malnutrition in this population. Stroke survivors with malnutrition might have a negative energy balance, which may affect their resistance to respiratory infections. Whether early immunomodulator use and nutrition support could improve the COVID-19 prognosis needs to be investigated.

Patients with previous stroke may experience a new cerebrovascular event with a different underlying pathophysiology. The importance of COVID-19 as a stroke trigger should not be underestimated. Two patients (33.3%) in our cohort experienced a new stroke event. Patients with COVID-19-associated stroke have shed light on specificities concerning clinical presentation and neuroimaging findings (56). Laboratory features of COVID-19-associated stroke include coagulopathy (i.e., elevated D-dimer), indicating the role of the hypercoagulable state (57). The pathogenesis and optimal management of ischemic stroke associated with COVID-19 still remain uncertain, but emerging evidence suggests that cytokine storm-triggered coagulopathy and endotheliopathy represent possible targetable mechanisms (58). Whether immediate prophylactic anticoagulation with low-molecular-weight heparin or full-intensity anticoagulation benefits patients with COVID-19-related ischemic stroke needs to be investigated.

We acknowledge the study limitations. First, our retrospective study is limited by the small sample size. Therefore, inferences drawn might be biased due to the small number of patients with previous stroke. In this case, four of six stroke patients at baseline were dependent on their caregiver, indicating a population with a severe burden of baseline disability. Our findings may not be generalized to stroke patients with mild baseline disability. However, our overall review of meta-analyses supports our retrospective study's findings, lending support to the association between previous stroke and unfavorable COVID-19 outcomes. Second, we only evaluated the association between previous stroke and short-term outcomes of COVID-19; future studies addressing the long-term outcomes are warranted. In terms of our overall review, we conducted a limited search in three main literature databases. Moreover, due to the lack of a definition of stroke or cerebrovascular disease in most included meta-analyses as well as in primary publications, we could not analyze different types of strokes or cerebrovascular pathology in the context of COVID-19 prognosis. Finally, the pooled estimated effect size in the most meta-analyses was not adjusted for confounding factors; further studies with individualized patient data meta-analyses might better address this topic.

## Conclusion

Our findings suggest that previous stroke seems to influence the course of COVID-19 infection; such patients are at high risk of unfavorable outcome and might benefit from specific COVID-19 prevention (e.g., priority vaccination) and early hospital treatment measures.

## Data Availability Statement

The raw data supporting the conclusions of this article will be made available by the authors, without undue reservation.

## Ethics Statement

The studies involving human participants were reviewed and approved by Fujian Medical University Union Hospital Ethics Committee. Written informed consent for participation was not required for this study in accordance with the national legislation and the institutional requirements.

## Author Contributions

HD has full access to all of the data in the study, takes responsibility for the integrity of the data, and the accuracy of the data analysis. HD and HH: study concept and design and drafting of the manuscript. HH, JC, SF, XC, XP, HLe, HLi, NL, and HD: acquisition, analysis, or interpretation of data. QY, PX, and YZ: critical revision of the manuscript for important intellectual content. PX: statistical analysis. All authors contributed to the article and approved the submitted version.

## Funding

This study was sponsored by the Key Clinical Specialty Discipline Construction Program of Fujian, P.R.C (No. 2100201).

## Conflict of Interest

The authors declare that the research was conducted in the absence of any commercial or financial relationships that could be construed as a potential conflict of interest.

## Publisher's Note

All claims expressed in this article are solely those of the authors and do not necessarily represent those of their affiliated organizations, or those of the publisher, the editors and the reviewers. Any product that may be evaluated in this article, or claim that may be made by its manufacturer, is not guaranteed or endorsed by the publisher.
