# Supplementary material for: Association Between Previous Stroke and Severe COVID-19: A Retrospective Cohort Study and an Overall Review of Meta-Analysis
Source: Front Neurol. 2022 Jul 12;13:922936. doi: 10.3389/fneur.2022.922936 (PMC9327441; doi:10.3389/fneur.2022.922936)
Supplement: Supplementary file 1 [file Data_Sheet_1.pdf]

Table S1: Primary publications in the included meta-analyses

	Barek et al	Del Sole et al.	Fang et al	Fernández et al.	Figliozzi et al.	Florez-Perdomo et al.	Gao et al	Katzenschlager et al.	Li et al.a	Li et al.b	Li SW et al	Patel et al.	Pranata et al.	Ramphul et al	Siepmann et al.	Singh et al.	Ssentongo et al.	Wang et al.a	Wang et al.b	Xu et al.	Yin et al.	Yu et al.	Zhang L et al	Zhou et al.
	(n=15)	(n=5)	(n=14)	(n=16)	(n=17)	(n=7)	(n=37)	(n=18)	(n=4)	(n=14)	(n=47)	(n=9)	(n=12)	(n=25)	(n=11)	(n=7)	(n=4)	(n=3)	(n=8)	(n=12)	(n=16)	(n=9)	(n=11)	(n=8)
Aggarwal S et al. [1]	★																							
Ahlstrom B et al. [2]											★													
Alguwaihes AM et al. [3]											★													
Almazeedi S et al. [4]																							★	
Alwafi H et al. [5]											★													
Aoun M et al. [6]											★													
Argenziano MG et al. [7]							★																	
Atkins JL et al. [8]											★									★				
Azarkar Z et al. [9]											★													
Bai T et al. [10]													★											
Bandera A et al. [11]											★													
Bhargava A et al. [12]														★										
Bonnet G et al. [13]											★													
Bushman D A et al. [14]											★													
Cai Q et al. [15]																							★	
Cao J et al. [16]				★	★			★					★		★		★		★					
Caro-Codon J et al. [17]											★													
Cen Y et al.																					★			

[18]																								
Chai C et al. [19]											★													
Chang MC et al. [20]																							★	
Chen F et al. [21]																				★				
Chen J et al. [22]																				★				
Chen R. et al. [23]								★																
Chen R et al. [24]																			★				★	
Chen SL et al. [25]																					★			
Chen T et al. [26]					★											★						★		
Chen T et al. [27]			★		★	★		★					★		★	★			★					★
Chen X et al. [28]	★													★									★	★
Chen x et al. [29]			★																					
Cheng K et al. [30]																					★			
Choi MH et al. [31]																				★				
Clift AK et al. [32]											★													
Cummins L et al. [33]											★													
Du R et al. [34]																							★	
Du R et al. [35]				★																				
Efros O et al. [36]											★													
El Aidaoui K et al. [37]														★										
Eskandar EN et al. [38]											★													

F.R.S.S.S.C.I et al. [39]											★													
Fan FSY et al. [40]											★													
Fang H et al. [41]																					★			
Fang XW et a. [42]			★																		★			
Feng Y et al. [43]	★			★	★		★		★	★					★	★					★		★	★
Ferguson J et al. [44]	★																							
Fu J et al. [45]							★																	
Galvez-Barron C et al. [46]											★													
Gonzalez-Fajardo JA et al. [47]											★													
Grasselli G et al. [48]						★																		
Graziani D et al. [49]											★													
Guan, WJ et al. [50]	★	★	★	★	★	★	★		★	★		★	★			★	★	★		★		★	★	★
Guo W et al. [51]																★								
Gupta A et al. [52]											★													
Huang J.et al.								★																
Han MF et al. [53]							★																	
He R et al. [54]	★																							
Hobbs ALV et al. [55]											★													
Hong KS et al. [56]	★																							
Hu H.et al.								★																

[57]																								
Hu L et al. [59]							★			★				★										
Hu L et al. [58]			★										★											
Huang H et al. [60]							★							★							★			
Huang Q et al. [61]	★						★																	
Huang R et al. [62]	★													★										
Hwang JM et al. [63]																				★				
Isik F et al. [64]											★													
Javanian M.et al. [65]								★																
Ji W et al. [66]														★										
Kelly JD et al. [67]											★													
Kummer BR et al. [68]											★													
Kutluhan MA et al. [69]							★																	
Kvale R et al. [70]											★													
Lee JH et al. [71]											★													
Lee JY et al. [72]														★										
Lei S et al. [73]	★			★			★								★									
Li J et al. [74]																			★					
li Q et al. [75]							★						★	★										
Li T et al. [76]							★							★										
Li Y et al.																						★		

[77]																								
Liang W et al. [78]	★																							
Liang WH et al. [79]															★									
Ling SF et al. [80]										★														
Liu J et al. [81]										★														
Liu Q et al. [82]						★																		
Liu SQ et al. [83]						★							★							★				
Liu Y et al. [84]			★	★																				
Lu H et al. [85]			★																					
Lu L et al. [86]						★																		
Lu Y et al. [87]										★														
Lugon JR et al. [88]										★														
Luo P et al. [89]					★																			
Luo XM et al. [90]												★												
Luo X et al. [91]							★																	
Lv YD et al. [92]																				★				
Lyu PJ et al. [93]	★					★																		
Maeda T et al. [94]						★																		
Magleby R et al. [95]																			★					
Mao L et al. [96]				★																	★			
Marques M et al. [97]										★														

Matsunaga N et al. [98]													★											
Miller J et al. [99]										★														
Mo P et al. [100]					★						★											★		
Muhammad R et al. [101]										★														
Nimkar A et al. [102]										★														
Ouattara E et al. [103]										★														
Panagides V et al. [104]										★														
Paranjpe I et al. [105]								★			★													
Perez-Guzman PN et al. [106]										★														
Pettit NN et al. [107]																			★					
Popov GT, et al. [108]													★											
Puebla Neira DA et al. [109]										★														
Qin C et al. [110]			★				★				★	★	★											★
Reilev M et al. [111]										★														
Romero-Sánchez CM et al. [112]							★														★			
Rossi L et al. [113]										★														
Ruan Q et al. [114]					★						★							★			★			
Semenzato L et al. [115]										★														
Shabrawishi M et al. [116]							★						★											

Sheshah E et al. [117]											★													
Shi Q. et al. [118]								★																
Shi S et al. [119]					★												★							
Shu Z et al. [120]										★											★			
Tomlins J et al. [121]				★				★											★					
Vogels Y et al. [122]											★													
Wan S et al. [123]										★														
Wang B et al. [124]											★													
Wang B et al. [125]														★										
Wang CZ et al. [126]							★																	
Wang, D et al. [127]		★		★	★	★	★		★	★		★		★	★	★		★				★	★	★
Wang, D et al. [128]							★						★											
Wang D. et al. [129]			★	★				★																
Wang F et al. [130]	★																							
Wang K et al. [131]								★																
Wang K et al [132]		★												★										
Wang L et al. [133]				★	★		★	★				★			★		★		★					
Wang L et al. [134]					★																			
Wang LW et al. [135]			★																					
Wang W et al. [136]							★																	
Wang Y et al.							★			★				★										

[137]																								
Wang Y et al. [138]			★				★			★			★											
Wei Y et al. [139]							★			★				★							★			
Wu J et al. [140]	★						★																	
Wu S et al. [141]							★																	
Xiong Set al. [142]										★														
Xiong TY et al. [143]																				★				
Xu XW et al. [144]															★			★				★		
Yuan M et al. [153]			★	★	★			★					★		★								★	
Yan X et al. [145]							★																	
Yan X et al. [146]				★																★				
Yang Q et al. [147]		★					★			★														
Yang X et al. [148]		★			★	★		★				★			★				★					
Yang Y et al. [149]																				★				
Yao CY et al. [150]																					★			
Yu C. et al. [152]								★						★										
Yu C et al. [151]										★														
Zagidullin NS et al. [154]											★													
Zerbo O et al. [155]											★													
Zhang GQ et al. [157]				★			★						★	★							★	★		

Zhang H et al. [158]							★																	
Zhang, JJ et al. [160]					★		★					★		★	★									★
Zhang J et al. [159]				★	★			★																
Zhang F.et al. [156]			★					★																
Zhang NN et al. [161]										★											★			
Zhang Q et al. [162]										★														
Zhang Y et al. [163]																					★			
Zhao C et al. [164]																					★			
Zhao M et al. [165]																				★				
Zhao XY et al. [167]						★																		
Zhao W et al. [166]			★											★										
Zheng F et al. [168]				★			★		★					★	★									★
Zhou Y et al. [170]					★																			
Zhou Y et al. [171]	★																							
Zhou F et al. [169]						★																		
Zou W et al. [172]							★																	

References:

[1] Aggarwal S, Garcia-Telles N, Aggarwal G, Lavie C, Lippi G, Henry BM. Clinical features, laboratory characteristics, and outcomes of patients hospitalized with coronavirus disease 2019 (COVID-19): Early report from the United States. Diagnosis (Berlin, Germany). 2020;7:91-6.https://doi.10.1515/dx-2020-0046.

[2] Ahlström B, Frithiof R, Hultström M, Larsson IM, Strandberg G, Lipcsey M. The swedish covid-19 intensive care cohort: Risk factors of ICU admission and ICU mortality. Acta anaesthesiologica Scandinavica. 2021;65:525-33.https://doi.10.1111/aas.13781.

[3] Alguwaihes AM, Al-Sofiani ME, Megdad M, Albader SS, Alsari MH, Alelayan A, et al. Diabetes and Covid-19 among hospitalized patients in Saudi Arabia: a single-centre retrospective study. Cardiovascular diabetology. 2020;19:205.https://doi.10.1186/s12933-020-01184-4.

[4] Almazeedi S, Al-Youha S, Jamal MH, Al-Haddad M, Al-Muhaini A, Al-Ghimlas F, et al. Characteristics, risk factors and outcomes among the first consecutive 1096 patients diagnosed with COVID-19 in Kuwait. EClinicalMedicine. 2020;24:100448.https://doi.10.1016/j.eclinm.2020.100448.

[5] Alwafi H, Naser AY, Qanash S, Brinji AS, Ghazawi MA, Alotaibi B, et al. Predictors of Length of Hospital Stay, Mortality, and Outcomes Among Hospitalised COVID-19 Patients in Saudi Arabia: A Cross-Sectional Study. Journal of multidisciplinary healthcare. 2021;14:839-52.https://doi.10.2147/jmdh.S304788.

[6] Aoun M, Khalil R, Mahfoud W, Fatfat H, Bou Khalil L, Alameddine R, et al. Age and multimorbidities as poor prognostic factors for COVID-19 in hemodialysis: a Lebanese national study. BMC nephrology. 2021;22:73.https://doi.10.1186/s12882-021-02270-9.

[7] Argenziano MG, Bruce SL, Slater CL, Tiao JR, Baldwin MR, Barr RG, et al. Characterization and clinical course of 1000 patients with coronavirus disease 2019 in New York: retrospective case series. BMJ (Clinical research ed). 2020;369:m1996.https://doi.10.1136/bmj.m1996.

[8] Atkins JL, Masoli JAH, Delgado J, Pilling LC, Kuo CL, Kuchel GA, et al. Preexisting Comorbidities Predicting COVID-19 and Mortality in the UK Biobank Community Cohort. The journals of gerontology Series A, Biological sciences and medical sciences. 2020;75:2224-30.https://doi.10.1093/gerona/glaa183.

[9] Azarkar Z, Salehiniya H, Kazemi T, Abbaszadeh H. Epidemiological, imaging, laboratory, and clinical characteristics and factors related to mortality in patients with COVID-19: a single-center study. Osong public health and research perspectives. 2021;12:169-76.https://doi.10.24171/j.phrp.2021.0012.

[10] Bai T, Tu S, Wei Y, Xiao L, Jin Y, Zhang L, et al. Clinical and Laboratory Factors Predicting the Prognosis of Patients with COVID-19: An Analysis of 127 Patients in Wuhan, China. Social Science Electronic Publishing.

[11] Bandera A, Nobili A, Tettamanti M, Harari S, Bosari S, Mannucci PM. Clinical factors associated with death in 3044 COVID-19 patients managed in internal medicine wards in Italy: comment. Internal and emergency medicine. 2022;17:299-302.https://doi.10.1007/s11739-021-02797-7.

[12] Bhargava A, Fukushima EA, Levine M, Zhao W, Tanveer F, Szpunar SM, et al. Predictors for Severe COVID-19 Infection. Clinical infectious diseases : an official publication of the Infectious Diseases Society of America. 2020;71:1962-8.https://doi.10.1093/cid/ciaa674.

[13] Bonnet G, Weizman O, Trimaille A, Pommier T, Cellier J, Geneste L, et al. Characteristics and outcomes of patients hospitalized for COVID-19 in France: The Critical COVID-19 France (CCF) study. Archives of cardiovascular diseases. 2021;114:352-63.https://doi.10.1016/j.acvd.2021.01.003.

[14] Bushman D, Davidson A, Pathela P, Greene SK, Weiss D, Reddy V, et al. Risk Factors for Death Among Hospitalized Patients Aged 21-64 Years Diagnosed with COVID-19-New York City, March 13-April 9, 2020. Journal of racial and ethnic health disparities. 2021:1-16.https://doi.10.1007/s40615-021-01098-1.

[15] Cai Q, Chen F, Wang T, Luo F, Liu X, Wu Q, et al. Obesity and COVID-19 Severity in a Designated Hospital in Shenzhen, China. Diabetes care. 2020;43:1392-8.https://doi.10.2337/dc20-0576.

[16] Cao J, Tu WJ, Cheng W, Yu L, Liu YK, Hu X, et al. Clinical Features and Short-term Outcomes of 102 Patients with Coronavirus

Disease 2019 in Wuhan, China. Clinical infectious diseases : an official publication of the Infectious Diseases Society of America. 2020;71:748-55.https://doi.10.1093/cid/ciaa243.

[17] Caro-Codón J, Rey JR, Buño A, Iniesta AM, Rosillo SO, Castrejon-Castrejon S, et al. Characterization of myocardial injury in a cohort of patients with SARS-CoV-2 infection. Medicina clinica. 2021;157:274-80.https://doi.10.1016/j.medcli.2021.02.001.

[18] Cen Y, Chen X, Shen Y, Zhang XH, Lei Y, Xu C, et al. Risk factors for disease progression in patients with mild to moderate coronavirus disease 2019-a multi-centre observational study. Clinical microbiology and infection : the official publication of the European Society of Clinical Microbiology and Infectious Diseases. 2020;26:1242-7.https://doi.10.1016/j.cmi.2020.05.041.

[19] Chai C, Feng X, Lu M, Li S, Chen K, Wang H, et al. One-year mortality and consequences of COVID-19 in cancer patients: A cohort study. IUBMB life. 2021;73:1244-56.https://doi.10.1002/iub.2536.

[20] Chang MC, Hwang JM, Jeon JH, Kwak SG, Park D, Moon JS. Fasting Plasma Glucose Level Independently Predicts the Mortality of Patients with Coronavirus Disease 2019 Infection: A Multicenter, Retrospective Cohort Study. Endocrinology and metabolism (Seoul, Korea). 2020;35:595-601.https://doi.10.3803/EnM.2020.719.

[21] Chen F, Sun W, Sun S, Li Z, Wang Z, Yu L. Clinical characteristics and risk factors for mortality among inpatients with COVID-19 in Wuhan, China. Clinical and translational medicine. 2020;10:e40.https://doi.10.1002/ctm2.40.

[22] Chen J, Bai H, Liu J, Chen G, Liao Q, Yang J, et al. Distinct Clinical Characteristics and Risk Factors for Mortality in Female Inpatients With Coronavirus Disease 2019 (COVID-19): A Sex-stratified, Large-scale Cohort Study in Wuhan, China. Clinical infectious diseases : an official publication of the Infectious Diseases Society of America. 2020;71:3188-95.https://doi.10.1093/cid/ciaa920.

[23] Chen R, Liang W, Jiang M, Guan W, Zhan C, Wang T, et al. Risk Factors of Fatal Outcome in Hospitalized Subjects With Coronavirus Disease 2019 From a Nationwide Analysis in China. Chest. 2020;158:97-105.https://doi.10.1016/j.chest.2020.04.010.

[24] Chen R, Sang L, Jiang M, Yang Z, Jia N, Fu W, et al. Longitudinal hematologic and immunologic variations associated with the progression of COVID-19 patients in China. The Journal of allergy and clinical immunology. 2020;146:89-100.https://doi.10.1016/j.jaci.2020.05.003.

[25] Chen SL, Feng HY, Xu H, Huang SS, Sun JF, Zhou L, et al. Patterns of Deterioration in Moderate Patients With COVID-19 From Jan 2020 to Mar 2020: A Multi-Center, Retrospective Cohort Study in China. Frontiers in medicine. 2020;7:567296.https://doi.10.3389/fmed.2020.567296.

[26] Chen T, Dai Z, Mo P, Li X, Ma Z, Song S, et al. Clinical Characteristics and Outcomes of Older Patients with Coronavirus Disease 2019 (COVID-19) in Wuhan, China: A Single-Centered, Retrospective Study. The journals of gerontology Series A, Biological sciences and medical sciences. 2020;75:1788-95.https://doi.10.1093/gerona/glaa089.

[27] Chen T, Wu D, Chen H, Yan W, Yang D, Chen G, et al. Clinical characteristics of 113 deceased patients with coronavirus disease 2019: retrospective study. BMJ (Clinical research ed). 2020;368:m1091.https://doi.10.1136/bmj.m1091.

[28] Chen X, Zhao B, Qu Y, Chen Y, Xiong J, Feng Y, et al. Detectable Serum Severe Acute Respiratory Syndrome Coronavirus 2 Viral Load (RNAemia) Is Closely Correlated With Drastically Elevated Interleukin 6 Level in Critically Ill Patients With Coronavirus Disease 2019. Clinical infectious diseases : an official publication of the Infectious Diseases Society of America. 2020;71:1937-42.https://doi.10.1093/cid/ciaa449.

[29] Chen X, Zheng F, Qing Y, Ding S, Yang D, Lei C, et al. Epidemiological and clinical features of 291 cases with coronavirus disease 2019 in areas adjacent to Hubei, China: a double-center observational study. medRxiv : the preprint server for health sciences. 2020:2020.03.03.20030353.https://doi.10.1101/2020.03.03.20030353.

[30] Cheng KB, Wei M, Shen H, Wu CM, Chen DC, Zhou XN, et al. Clinical characteristics of 463 patients with common and severe type coronavirus disease 2019. Shanghai Med J. 2020;43:224-32.https://doi.10.19842/j.cnki.issn.0253-9934.2020.04.008.

[31] Choi MH, Ahn H, Ryu HS, Kim BJ, Jang J, Jung M, et al. Clinical Characteristics and Disease Progression in Early-Stage COVID-19 Patients in South Korea. Journal of clinical medicine. 2020;9.https://doi.10.3390/jcm9061959.

[32] Clift AK, Coupland CAC, Keogh RH, Diaz-Ordaz K, Williamson E, Harrison EM, et al. Living risk prediction algorithm (QCOVID) for risk of hospital admission and mortality from coronavirus 19 in adults: national derivation and validation cohort

study. BMJ (Clinical research ed). 2020;371:m3731.https://doi.10.1136/bmj.m3731.

[33] Cummins L, Ebyarimpa I, Cheetham N, Tzortziou Brown V, Brennan K, Panovska-Griffiths J. Factors associated with COVID-19 related hospitalisation, critical care admission and mortality using linked primary and secondary care data. Influenza and other respiratory viruses. 2021;15:577-88.https://doi.10.1111/irv.12864.

[34] Du RH, Liang LR, Yang CQ, Wang W, Cao TZ, Li M, et al. Predictors of mortality for patients with COVID-19 pneumonia caused by SARS-CoV-2: a prospective cohort study. The European respiratory journal. 2020;55.https://doi.10.1183/13993003.00524-2020.

[35] Du RH, Liu LM, Yin W, Wang W, Guan LL, Yuan ML, et al. Hospitalization and Critical Care of 109 Decedents with COVID-19 Pneumonia in Wuhan, China. Annals of the American Thoracic Society. 2020;17:839-46.https://doi.10.1513/AnnalsATS.202003-225OC.

[36] Efros O, Barda N, Meisel E, Leibowitz A, Fardman A, Rahav G, et al. Myocardial injury in hospitalized patients with COVID-19 infection-Risk factors and outcomes. PloS one. 2021;16:e0247800.https://doi.10.1371/journal.pone.0247800.

[37] El Aidaoui K, Haoudar A, Khalis M, Kantri A, Ziati J, El Ghanmi A, et al. Predictors of Severity in Covid-19 Patients in Casablanca, Morocco. Cureus. 2020;12:e10716.https://doi.10.7759/cureus.10716.

[38] Eskandar EN, Altschul DJ, de la Garza Ramos R, Cezayirli P, Unda SR, Benton J, et al. Neurologic Syndromes Predict Higher In-Hospital Mortality in COVID-19. Neurology. 2021;96:e1527-e38.https://doi.10.1212/wnl.0000000000011356.

[39] F.R.S.S.S.C.I (2020) consortium c. Severity of COVID-19 and survival in patients with rheumatic and inflammatory diseases: data from the French RMD COVID-19 cohort of 694 patients. Annals of the rheumatic diseases. 2020;80:527-38.https://doi.10.1136/annrheumdis-2020-218310.

[40] Fan FSY, Yip TCF, Yiu B, Lam B, Au L, Lau AY, et al. Neurological diseases and risk of mortality in patients with COVID-19 and SARS: a territory-wide study in Hong Kong. Journal of neurology, neurosurgery, and psychiatry. 2021;92:1356-8.https://doi.10.1136/jnnp-2021-326286.

[41] Fang H, Liu Q, Xi M, Xiong D, He J, Luo P, et al. Impact of comorbidities on clinical prognosis in 1280 patients with different types of COVID-19. Journal of investigative medicine : the official publication of the American Federation for Clinical Research. 2021;69:75-85.https://doi.10.1136/jim-2020-001555.

[42] Fang X, Mei Q, Yang T, Zhang L, Yang Y, Wang Y, et al. Clinical characteristics and treatment strategies of 79 patients with COVID-19. Chinese Pharmacological Bulletin. 2020;36:453-9.https://doi.https://doi.10.3969/j.issn.1001-1978.2020.04.002.

[43] Feng Y, Ling Y, Bai T, Xie Y, Huang J, Li J, et al. COVID-19 with Different Severities: A Multicenter Study of Clinical Features. American journal of respiratory and critical care medicine. 2020;201:1380-8.https://doi.10.1164/rccm.202002-0445OC.

[44] Ferguson J, Rosser JI, Quintero O, Scott J, Subramanian A, Gumma M, et al. Characteristics and Outcomes of Coronavirus Disease Patients under Nonsurge Conditions, Northern California, USA, March-April 2020. Emerging infectious diseases. 2020;26:1679-85.https://doi.10.3201/eid2608.201776.

[45] Fu J, Huang PP, Zhang S, Yao QD, Han R, Liu HF, et al. The value of serum amyloid A for predicting the severity and recovery of COVID-19. Experimental and therapeutic medicine. 2020;20:3571-7.https://doi.10.3892/etm.2020.9114.

[46] Gálvez-Barrón C, Arroyo-Huidobro M, Miňarro A, Añaños G, Chamero A, Martín M, et al. COVID-19: Clinical Presentation and Prognostic Factors of Severe Disease and Mortality in the Oldest-Old Population: A Cohort Study. Gerontology. 2022;68:30-43.https://doi.10.1159/000515159.

[47] Gonzalez-Fajardo JA, Ansuategui M, Romero C, Comanges A, Gómez-Arbeláez D, Ibarra G, et al. Mortality of COVID-19 patients with vascular thrombotic complications. Medicina clinica (English ed). 2021;156:112-7.https://doi.10.1016/j.medcle.2020.10.008.

[48] Grasselli G, Zangrillo A, Zanella A, Antonelli M, Cabrini L, Castelli A, et al. Baseline Characteristics and Outcomes of 1591 Patients Infected With SARS-CoV-2 Admitted to ICUs of the Lombardy Region, Italy. Jama. 2020;323:1574-81.https://doi.10.1001/jama.2020.5394.

[49] Graziani D, Soriano JB, Del Rio-Bermudez C, Morena D, Díaz T, Castillo M, et al. Characteristics and Prognosis of COVID-19

in Patients with COPD. Journal of clinical medicine. 2020;9.https://doi.10.3390/jcm9103259.

[50] Guan WJ, Ni ZY, Hu Y, Liang WH, Ou CQ, He JX, et al. Clinical Characteristics of Coronavirus Disease 2019 in China. The New England journal of medicine. 2020;382:1708-20.https://doi.10.1056/NEJMoa2002032.

[51] Guo W, Li M, Dong Y, Zhou H, Zhang Z, Tian C, et al. Diabetes is a risk factor for the progression and prognosis of COVID-19. Diabetes/metabolism research and reviews. 2020:e3319.https://doi.10.1002/dmrr.3319.

[52] Gupta A, Madhavan MV, Poterucha TJ, DeFilippis EM, Hennessey JA, Redfors B, et al. Association between antecedent statin use and decreased mortality in hospitalized patients with COVID-19. Nature communications. 2021;12:1325.https://doi.10.1038/s41467-021-21553-1.

[53] Han M, Xu M, Zhang Y, Liu Z, Li S, He T, et al. Assessing SARS-CoV-2 RNA levels and lymphocyte/T cell counts in COVID-19 patients revealed initial immune status as a major determinant of disease severity. Medical microbiology and immunology. 2020;209:657-68.https://doi.10.1007/s00430-020-00693-z.

[54] He R, Lu Z, Zhang L, Fan T, Xiong R, Shen X, et al. The clinical course and its correlated immune status in COVID-19 pneumonia. Journal of clinical virology : the official publication of the Pan American Society for Clinical Virology. 2020;127:104361.https://doi.10.1016/j.jcv.2020.104361.

[55] Hobbs ALV, Turner N, Omer I, Walker MK, Beaulieu RM, Sheikh M, et al. Risk factors for mortality and progression to severe COVID-19 disease in the Southeast region in the United States: A report from the SEUS Study Group. Infection control and hospital epidemiology. 2021;42:1464-72.https://doi.10.1017/ice.2020.1435.

[56] Hong KS, Lee KH, Chung JH, Shin KC, Choi EY, Jin HJ, et al. Clinical Features and Outcomes of 98 Patients Hospitalized with SARS-CoV-2 Infection in Daegu, South Korea: A Brief Descriptive Study. Yonsei medical journal. 2020;61:431-7.https://doi.10.3349/ymj.2020.61.5.431.

[57] Hu H, Yao N, Qiu Y. Comparing Rapid Scoring Systems in Mortality Prediction of Critically Ill Patients With Novel Coronavirus Disease. Academic emergency medicine : official journal of the Society for Academic Emergency Medicine. 2020;27:461-8.https://doi.10.1111/acem.13992.

[58] Hu L, Chen S, Fu Y, Gao Z, Long H, Ren H-w, et al. Risk Factors Associated with Clinical Outcomes in 323 COVID-19 Patients in Wuhan, China. medRxiv : the preprint server for health sciences. 2020:2020.03.25.20037721.https://doi.10.1101/2020.03.25.20037721.

[59] Hu L, Chen S, Fu Y, Gao Z, Long H, Ren HW, et al. Risk Factors Associated With Clinical Outcomes in 323 Coronavirus Disease 2019 (COVID-19) Hospitalized Patients in Wuhan, China. Clinical infectious diseases : an official publication of the Infectious Diseases Society of America. 2020;71:2089-98.https://doi.10.1093/cid/ciaa539.

[60] Huang H, Song B, Xu Z, Jiao Y, Huang L, Zhao P, et al. Predictors of Coronavirus Disease 2019 Severity: A Retrospective Study of 64 Cases. Japanese journal of infectious diseases. 2021;74:54-60.https://doi.10.7883/yoken.JJID.2020.298.

[61] Huang Q, Deng X, Li Y, Sun X, Chen Q, Xie M, et al. Clinical characteristics and drug therapies in patients with the common-type coronavirus disease 2019 in Hunan, China. International journal of clinical pharmacy. 2020;42:837-45.https://doi.10.1007/s11096-020-01031-2.

[62] Huang R, Zhu L, Xue L, Liu L, Yan X, Wang J, et al. Clinical findings of patients with coronavirus disease 2019 in Jiangsu province, China: A retrospective, multi-center study. PLoS neglected tropical diseases. 2020;14:e0008280.https://doi.10.1371/journal.pntd.0008280.

[63] Hwang JM, Kim JH, Park JS, Chang MC, Park D. Neurological diseases as mortality predictive factors for patients with COVID-19: a retrospective cohort study. Neurological sciences : official journal of the Italian Neurological Society and of the Italian Society of Clinical Neurophysiology. 2020;41:2317-24.https://doi.10.1007/s10072-020-04541-z.

[64] Işık F, Çap M, Akyüz A, Bilge Ö, Aslan B, İnci Ü, et al. The effect of resistant hypertension on in-hospital mortality in patients hospitalized with COVID-19. Journal of human hypertension. 2021:1-6.https://doi.10.1038/s41371-021-00591-8.

[65] Javanian M, Bayani M, Shokri M, Sadeghi-Haddad-Zavareh M, Babazadeh A, Yeganeh B, et al. Clinical and laboratory findings from patients with COVID-19 pneumonia in Babol North of Iran: a retrospective cohort study. Romanian journal of internal

medicine = Revue roumaine de medecine interne. 2020;58:161-7.https://doi.10.2478/rjim-2020-0013.

[66] Ji W, Huh K, Kang M, Hong J, Bae GH, Lee R, et al. Effect of Underlying Comorbidities on the Infection and Severity of COVID-19 in Korea: a Nationwide Case-Control Study. Journal of Korean medical science. 2020;35:e237.https://doi.10.3346/jkms.2020.35.e237.

[67] Kelly JD, Bravata DM, Bent S, Wray CM, Leonard SJ, Boscardin WJ, et al. Association of Social and Behavioral Risk Factors With Mortality Among US Veterans With COVID-19. JAMA network open. 2021;4:e2113031.https://doi.10.1001/jamanetworkopen.2021.13031.

[68] Kummer BR, Klang E, Stein LK, Dhamoon MS, Jetté N. History of Stroke Is Independently Associated With In-Hospital Death in Patients With COVID-19. Stroke. 2020;51:3112-4.https://doi.10.1161/strokeaha.120.030685.

[69] Kutluhan MA, Taş A, Şahin A, Ürkmez A, Topaktas R, Ataç Ö, et al. Assessment of clinical features and renal functions in Coronavirus disease-19: A retrospective analysis of 96 patients. International journal of clinical practice. 2020;74:e13636.https://doi.10.1111/ijcp.13636.

[70] Kvåle R, Bønaa KH, Forster R, Gravningen K, Júlíusson PB, Myklebust T. Does a history of cardiovascular disease or cancer affect mortality after SARS-CoV-2 infection? Tidsskrift for den Norske laegeforening : tidsskrift for praktisk medicin, ny raekke. 2021;140.https://doi.10.4045/tidsskr.20.0956.

[71] Lee JH, Hwang YM, Cho Y, Oh IY. Prognostic impact of atrial fibrillation in patients with severe acute respiratory syndrome coronavirus 2 infection. Medicine. 2021;100:e26993.https://doi.10.1097/md.0000000000026993.

[72] Lee JY, Hong SW, Hyun M, Park JS, Lee JH, Suh YS, et al. Epidemiological and clinical characteristics of coronavirus disease 2019 in Daegu, South Korea. International journal of infectious diseases : IJID : official publication of the International Society for Infectious Diseases. 2020;98:462-6.https://doi.10.1016/j.ijid.2020.07.017.

[73] Lei S, Jiang F, Su W, Chen C, Chen J, Mei W, et al. Clinical characteristics and outcomes of patients undergoing surgeries during the incubation period of COVID-19 infection. EClinicalMedicine. 2020;21:100331.https://doi.10.1016/j.eclinm.2020.100331.

[74] Li J, Wang X, Chen J, Zhang H, Deng A. Association of Renin-Angiotensin System Inhibitors With Severity or Risk of Death in Patients With Hypertension Hospitalized for Coronavirus Disease 2019 (COVID-19) Infection in Wuhan, China. JAMA cardiology. 2020;5:825-30.https://doi.10.1001/jamacardio.2020.1624.

[75] Li Q, Ling Y, Zhang J, Li W, Chen L. Clinical Characteristics of SARS-CoV-2 Infections Involving 325 Hospitalized Patients outside Wuhan. 2020.https://doi.10.21203/rs.3.rs-18699/v1.

[76] Li T, Lu L, Zhang W, Tao Y, Wang L, Bao J, et al. Clinical characteristics of 312 hospitalized older patients with COVID-19 in Wuhan, China. Archives of gerontology and geriatrics. 2020;91:104185.https://doi.10.1016/j.archger.2020.104185.

[77] Li Y, Li M, Wang M, Zhou Y, Chang J, Xian Y, et al. Acute cerebrovascular disease following COVID-19: a single center, retrospective, observational study. Stroke and vascular neurology. 2020;5:279-84.https://doi.10.1136/svn-2020-000431.

[78] Liang W, Liang H, Ou L, Chen B, Chen A, Li C, et al. Development and Validation of a Clinical Risk Score to Predict the Occurrence of Critical Illness in Hospitalized Patients With COVID-19. JAMA internal medicine. 2020;180:1081-9.https://doi.10.1001/jamainternmed.2020.2033.

[79] Liang WH, Guan WJ, Li CC, Li YM, Liang HR, Zhao Y, et al. Clinical characteristics and outcomes of hospitalised patients with COVID-19 treated in Hubei (epicentre) and outside Hubei (non-epicentre): a nationwide analysis of China. The European respiratory journal. 2020;55.https://doi.10.1183/13993003.00562-2020.

[80] Ling SF, Broad E, Murphy R, Pappachan JM, Pardesi-Newton S, Kong MF, et al. High-Dose Cholecalciferol Booster Therapy is Associated with a Reduced Risk of Mortality in Patients with COVID-19: A Cross-Sectional Multi-Centre Observational Study. Nutrients. 2020;12.https://doi.10.3390/nu12123799.

[81] Liu J, Zhang S, Dong X, Li Z, Xu Q, Feng H, et al. Corticosteroid treatment in severe COVID-19 patients with acute respiratory distress syndrome. The Journal of clinical investigation. 2020;130:6417-28.https://doi.10.1172/jci140617.

[82] Liu Q, Dai Y, Feng M, Wang X, Liang W, Yang F. Associations between serum amyloid A, interleukin-6, and COVID-19: A cross-sectional study. Journal of clinical laboratory analysis. 2020;34:e23527.https://doi.10.1002/jcla.23527.

[83] Liu S, Luo H, Wang Y, Cuevas LE, Wang D, Ju S, et al. Clinical characteristics and risk factors of patients with severe COVID-19 in Jiangsu province, China: a retrospective multicentre cohort study. BMC infectious diseases. 2020;20:584.https://doi.10.1186/s12879-020-05314-x.

[84] Liu Y, Sun W, Li J, Chen L, Wang Y, Zhang L, et al. Clinical features and progression of acute respiratory distress syndrome in coronavirus disease 2019. medRxiv : the preprint server for health sciences. 2020:2020.02.17.20024166.https://doi.10.1101/2020.02.17.20024166.

[85] Lu H, Ai J, Shen Y, Li Y, Li T, Zhou X, et al. A descriptive study of the impact of diseases control and prevention on the epidemics dynamics and clinical features of SARS-CoV-2 outbreak in Shanghai, lessons learned for metropolis epidemics prevention. medRxiv : the preprint server for health sciences. 2020:2020.02.19.20025031.https://doi.10.1101/2020.02.19.20025031.

[86]Lu L, Xiong W, Liu D, Liu J, Yang D, Li N, et al. New onset acute symptomatic seizure and risk factors in coronavirus disease 2019: A retrospective multicenter study. Epilepsia. 2020;61:e49-e53.https://doi.10.1111/epi.16524.

[87] Lu Y, Jiao Y, Graham DJ, Wu Y, Wang J, Menis M, et al. Risk Factors for COVID-19 Deaths Among Elderly Nursing Home Medicare Beneficiaries in the Prevaccine Period. The Journal of infectious diseases. 2022;225:567-77.https://doi.10.1093/infdis/jiab515.

[88] Lugon JR, Neves P, Pio-Abreu A, do Nascimento MM, Sesso R. Evaluation of central venous catheter and other risk factors for mortality in chronic hemodialysis patients with COVID-19 in Brazil. International urology and nephrology. 2022;54:193-9.https://doi.10.1007/s11255-021-02920-9.

[89] Luo P, Liu Y, Qiu L, Liu X, Liu D, Li J. Tocilizumab treatment in COVID-19: A single center experience. Journal of medical virology. 2020;92:814-8.https://doi.https://doi.org/10.1002/jmv.25801.

[90] Luo X, Zhou W, Yan X, Guo T, Wang B, Xia H, et al. Prognostic Value of C-Reactive Protein in Patients With Coronavirus 2019. Clinical infectious diseases : an official publication of the Infectious Diseases Society of America. 2020;71:2174-9.https://doi.10.1093/cid/ciaa641.

[91] Luo X, Zhou W, Yan X, Guo T, Wang B, Xia H, et al. Prognostic Value of C-Reactive Protein in Patients With Coronavirus 2019. Clinical Infectious Diseases. 2020;71:2174-9.https://doi.10.1093/cid/ciaa641.

[92] Lv Y. Gansu Medical Journal. 2020:39(03): 247250

[93] Lyu P, Liu X, Zhang R, Shi L, Gao J. The Performance of Chest CT in Evaluating the Clinical Severity of COVID-19 Pneumonia: Identifying Critical Cases Based on CT Characteristics. Investigative radiology. 2020;55:412-21.https://doi.10.1097/rli.0000000000000689.

[94] Maeda T, Obata R, Rizk DD, Kuno T. The association of interleukin-6 value, interleukin inhibitors, and outcomes of patients with COVID-19 in New York City. Journal of medical virology. 2021;93:463-71.https://doi.10.1002/jmv.26365.

[95] Magleby R, Westblade LF, Trzebucki A, Simon MS, Rajan M, Park J, et al. Impact of Severe Acute Respiratory Syndrome Coronavirus 2 Viral Load on Risk of Intubation and Mortality Among Hospitalized Patients With Coronavirus Disease 2019. Clinical infectious diseases : an official publication of the Infectious Diseases Society of America. 2021;73:e4197-e205.https://doi.10.1093/cid/ciaa851.

[96] Mao L, Jin H, Wang M, Hu Y, Chen S, He Q, et al. Neurologic Manifestations of Hospitalized Patients With Coronavirus Disease 2019 in Wuhan, China. JAMA neurology. 2020;77:683-90.https://doi.10.1001/jamaneurol.2020.1127.

[97] Marquès M, Correig E, Ibarretxe D, Anoro E, Antonio Arroyo J, Jericó C, et al. Long-term exposure to PM(10) above WHO guidelines exacerbates COVID-19 severity and mortality. Environment international. 2022;158:106930.https://doi.10.1016/j.envint.2021.106930.

[98] Matsunaga N, Hayakawa K, Terada M, Ohtsu H, Asai Y, Tsuzuki S, et al. Clinical Epidemiology of Hospitalized Patients With Coronavirus Disease 2019 (COVID-19) in Japan: Report of the COVID-19 Registry Japan. Clinical infectious diseases : an official publication of the Infectious Diseases Society of America. 2021;73:e3677-e89.https://doi.10.1093/cid/ciaa1470.

[99] Miller J, Fadel RA, Tang A, Perrotta G, Herc E, Soman S, et al. The Impact of Sociodemographic Factors, Comorbidities, and Physiologic Responses on 30-Day Mortality in Coronavirus Disease 2019 (COVID-19) Patients in Metropolitan Detroit. Clinical

infectious diseases : an official publication of the Infectious Diseases Society of America. 2021;72:e704-e10.https://doi.10.1093/cid/ciaa1420.

[100] Mo P, Xing Y, Xiao Y, Deng L, Zhao Q, Wang H, et al. Clinical Characteristics of Refractory Coronavirus Disease 2019 in Wuhan, China. Clinical infectious diseases : an official publication of the Infectious Diseases Society of America. 2021;73:e4208-e13.https://doi.10.1093/cid/ciaa270.

[101] Muhammad R, Ogunti R, Ahmad B, Munawar M, Donaldson S, Sumon M, et al. Clinical Characteristics and Predictors of Mortality in Minority Patients Hospitalized with COVID-19 Infection. Journal of racial and ethnic health disparities. 2022;9:335-45.https://doi.10.1007/s40615-020-00961-x.

[102] Nimkar A, Naaraayan A, Hasan A, Pant S, Durdevic M, Suarez CN, et al. Incidence and Risk Factors for Acute Kidney Injury and Its Effect on Mortality in Patients Hospitalized From COVID-19. Mayo Clinic proceedings Innovations, quality & outcomes. 2020;4:687-95.https://doi.10.1016/j.mayocpiqo.2020.07.003.

[103] Ouattara E, Bruandet A, Borde A, Lenne X, Binder-Foucard F, Le-Bourhis-Zaimi M, et al. Risk factors of mortality among patients hospitalised with COVID-19 in a critical care or hospital care unit: analysis of the French national medicoadministrative database. BMJ open respiratory research. 2021;8.https://doi.10.1136/bmjresp-2021-001002.

[104] Panagides V, Vincent F, Weizman O, Jonveaux M, Trimaille A, Pommier T, et al. History of heart failure in patients with coronavirus disease 2019: Insights from a French registry. Archives of cardiovascular diseases. 2021;114:415-25.https://doi.10.1016/j.acvd.2021.04.003.

[105] Paranjpe I, Russak AJ, De Freitas JK, Lala A, Miotto R, Vaid A, et al. Retrospective cohort study of clinical characteristics of 2199 hospitalised patients with COVID-19 in New York City. BMJ open. 2020;10:e040736.https://doi.10.1136/bmjopen-2020-040736.

[106] Perez-Guzman PN, Daunt A, Mukherjee S, Crook P, Forlano R, Kont MD, et al. Clinical Characteristics and Predictors of Outcomes of Hospitalized Patients With Coronavirus Disease 2019 in a Multiethnic London National Health Service Trust: A Retrospective Cohort Study. Clinical infectious diseases : an official publication of the Infectious Diseases Society of America. 2021;73:e4047-e57.https://doi.10.1093/cid/ciaa1091.

[107] Pettit NN, MacKenzie EL, Ridgway JP, Pursell K, Ash D, Patel B, et al. Obesity is Associated with Increased Risk for Mortality Among Hospitalized Patients with COVID-19. Obesity (Silver Spring, Md). 2020;28:1806-10.https://doi.10.1002/oby.22941.

[108] Popov GT, Baymakova M, Vaseva V, Kundurzhiev T, Mutafchiyski V. Clinical Characteristics of Hospitalized Patients with COVID-19 in Sofia, Bulgaria. Vector borne and zoonotic diseases (Larchmont, NY). 2020;20:910-5.https://doi.10.1089/vbz.2020.2679.

[109] Puebla Neira DA, Watts A, Seashore J, Duarte A, Nishi SP, Polychronopoulou E, et al. Outcomes of Patients with COPD Hospitalized for Coronavirus Disease 2019. Chronic obstructive pulmonary diseases (Miami, Fla). 2021;8:517-27.https://doi.10.15326/jcopdf.2021.0245.

[110] Qin C, Zhou L, Hu Z, Zhang S, Yang S, Tao Y, et al. Dysregulation of Immune Response in Patients With Coronavirus 2019 (COVID-19) in Wuhan, China. Clinical infectious diseases : an official publication of the Infectious Diseases Society of America. 2020;71:762-8.https://doi.10.1093/cid/ciaa248.

[111] Reilev M, Kristensen KB, Pottegård A, Lund LC, Hallas J, Ernst MT, et al. Characteristics and predictors of hospitalization and death in the first 11 122 cases with a positive RT-PCR test for SARS-CoV-2 in Denmark: a nationwide cohort. International journal of epidemiology. 2020;49:1468-81.https://doi.10.1093/ije/dyaa140.

[112] Romero-Sánchez CM, Díaz-Maroto I, Fernández-Díaz E, Sánchez-Larsen Á, Layos-Romero A, García-García J, et al. Neurologic manifestations in hospitalized patients with COVID-19: The ALBACOVID registry. Neurology. 2020;95:e1060-e70.https://doi.10.1212/wnl.0000000000009937.

[113] Rossi L, Malagoli A, Biagi A, Zanni A, Sticozzi C, Comastri G, et al. Renin-angiotensin system inhibitors and mortality in patients with COVID-19. Infection. 2021;49:287-94.https://doi.10.1007/s15010-020-01550-0.

[114] Ruan Q, Yang K, Wang W, Jiang L, Song J. Clinical predictors of mortality due to COVID-19 based on an analysis of data of 150 patients from Wuhan, China. Intensive care medicine. 2020;46:846-8.https://doi.10.1007/s00134-020-05991-x.

[115] Semenzato L, Botton J, Drouin J, Cuenot F, Dray-Spira R, Weill A, et al. Chronic diseases, health conditions and risk of COVID-19-related hospitalization and in-hospital mortality during the first wave of the epidemic in France: a cohort study of 66 million people. The Lancet regional health Europe. 2021;8:100158.https://doi.10.1016/j.lanepe.2021.100158.

[116] Shabrawishi M, Al-Gethamy MM, Naser AY, Ghazawi MA, Alsharif GF, Obaid EF, et al. Clinical, radiological and therapeutic characteristics of patients with COVID-19 in Saudi Arabia. PloS one. 2020;15:e0237130.https://doi.10.1371/journal.pone.0237130.

[117] Sheshah E, Sabico S, Albakr RM, Sultan AA, Alghamdi KS, Al Madani K, et al. Prevalence of diabetes, management and outcomes among Covid-19 adult patients admitted in a specialized tertiary hospital in Riyadh, Saudi Arabia. Diabetes research and clinical practice. 2021;172:108538.https://doi.10.1016/j.diabres.2020.108538.

[118] Shi Q, Zhang X, Jiang F, Zhang X, Hu N, Bimu C, et al. Clinical Characteristics and Risk Factors for Mortality of COVID-19 Patients With Diabetes in Wuhan, China: A Two-Center, Retrospective Study. Diabetes care. 2020;43:1382-91.https://doi.10.2337/dc20-0598.

[119] Shi S, Qin M, Shen B, Cai Y, Liu T, Yang F, et al. Association of Cardiac Injury With Mortality in Hospitalized Patients With COVID-19 in Wuhan, China. JAMA cardiology. 2020;5:802-10.https://doi.10.1001/jamacardio.2020.0950.

[120] Shu Z, Zhou Y, Chang K, Liu J, Min X, Zhang Q, et al. Clinical features and the traditional Chinese medicine therapeutic characteristics of 293 COVID-19 inpatient cases. Frontiers of medicine. 2020;14:760-75.https://doi.10.1007/s11684-020-0803-8.

[121] Tomlins J, Hamilton F, Gunning S, Sheehy C, Moran E, MacGowan A. Clinical features of 95 sequential hospitalised patients with novel coronavirus 2019 disease (COVID-19), the first UK cohort. The Journal of infection. 2020;81:e59-e61.https://doi.10.1016/j.jinf.2020.04.020.

[122] Vogels Y, Pouwels S, van Oers J, Ramnarain D. Characteristics and Risk Factors Associated With Mortality in Critically Ill Patients With COVID-19. Cureus. 2021;13:e14442.https://doi.10.7759/cureus.14442.

[123] Wan S, Xiang Y, Fang W, Zheng Y, Li B, Hu Y, et al. Clinical features and treatment of COVID-19 patients in northeast Chongqing. Journal of medical virology. 2020;92:797-806.https://doi.10.1002/jmv.25783.

[124] Wang B, Glicksberg BS, Nadkarni GN, Vashishth D. Evaluation and management of COVID-19-related severity in people with type 2 diabetes. BMJ open diabetes research & care. 2021;9.https://doi.10.1136/bmjdrc-2021-002299.

[125] Wang B, Wang Z, Zhao J, Zeng X, Wu M, Wang S, et al. Epidemiological and clinical course of 483 patients with COVID-19 in Wuhan, China: a single-center, retrospective study from the mobile cabin hospital. European journal of clinical microbiology & infectious diseases : official publication of the European Society of Clinical Microbiology. 2020;39:2309-15.https://doi.10.1007/s10096-020-03927-3.

[126] Wang C, Deng R, Gou L, Fu Z, Zhang X, Shao F, et al. Preliminary study to identify severe from moderate cases of COVID-19 using combined hematology parameters. Annals of translational medicine. 2020;8:593.https://doi.10.21037/atm-20-3391.

[127] Wang D, Hu B, Hu C, Zhu F, Liu X, Zhang J, et al. Clinical Characteristics of 138 Hospitalized Patients With 2019 Novel Coronavirus-Infected Pneumonia in Wuhan, China. Jama. 2020;323:1061-9.https://doi.10.1001/jama.2020.1585.

[128] Wang D, Li R, Wang J, Jiang Q, Gao C, Yang J, et al. Correlation analysis between disease severity and clinical and biochemical characteristics of 143 cases of COVID-19 in Wuhan, China: a descriptive study. BMC infectious diseases. 2020;20:519.https://doi.10.1186/s12879-020-05242-w.

[129] Wang D, Yin Y, Hu C, Liu X, Zhang X, Zhou S, et al. Clinical course and outcome of 107 patients infected with the novel coronavirus, SARS-CoV-2, discharged from two hospitals in Wuhan, China. Critical care (London, England). 2020;24:188.https://doi.10.1186/s13054-020-02895-6.

[130] Wang F, Yang Y, Dong K, Yan Y, Zhang S, Ren H, et al. CLINICAL CHARACTERISTICS OF 28 PATIENTS WITH DIABETES AND COVID-19 IN WUHAN, CHINA. Endocrine practice : official journal of the American College of Endocrinology and the American Association of Clinical Endocrinologists. 2020;26:668-74.https://doi.10.4158/ep-2020-0108.

[131] Wang K, Zuo P, Liu Y, Zhang M, Zhao X, Xie S, et al. Clinical and Laboratory Predictors of In-hospital Mortality in Patients With Coronavirus Disease-2019: A Cohort Study in Wuhan, China. Clinical Infectious Diseases. 2020;71:2079-

88.https://doi.10.1093/cid/ciaa538.

[132] Wang K, Zuo P, Liu Y, Zhang M, Zhao X, Xie S, et al. Clinical and Laboratory Predictors of In-hospital Mortality in Patients With Coronavirus Disease-2019: A Cohort Study in Wuhan, China. Clinical infectious diseases : an official publication of the Infectious Diseases Society of America. 2020;71:2079-88.https://doi.10.1093/cid/ciaa538.

[133] Wang L, He W, Yu X, Hu D, Bao M, Liu H, et al. Coronavirus disease 2019 in elderly patients: Characteristics and prognostic factors based on 4-week follow-up. The Journal of infection. 2020;80:639-45.https://doi.10.1016/j.jinf.2020.03.019.

[134] Wang L, Li X, Chen H, Yan S, Li D, Li Y, et al. Coronavirus Disease 19 Infection Does Not Result in Acute Kidney Injury: An Analysis of 116 Hospitalized Patients from Wuhan, China. American journal of nephrology. 2020;51:343-8.https://doi.10.1159/000507471.

[135] Wang L, Li X, Chen H, Yan S, Li Y, Li D, et al. SARS-CoV-2 infection does not significantly cause acute renal injury: an analysis of 116 hospitalized patients with COVID-19 in a single hospital, Wuhan, China. medRxiv : the preprint server for health sciences. 2020:2020.02.19.20025288.https://doi.10.1101/2020.02.19.20025288.

[136] Wang W, Xin C, Xiong Z, Yan X, Cai Y, Zhou K, et al. Clinical Characteristics and Outcomes of 421 Patients With Coronavirus Disease 2019 Treated in a Mobile Cabin Hospital. Chest. 2020;158:939-46.https://doi.10.1016/j.chest.2020.05.515.

[137] Wang Y, Liao B, Guo Y, Li F, Lei C, Zhang F, et al. Clinical Characteristics of Patients Infected With the Novel 2019 Coronavirus (SARS-Cov-2) in Guangzhou, China. Open forum infectious diseases. 2020;7:ofaa187.https://doi.10.1093/ofid/ofaa187.

[138] Wang Y, Zhou Y, Yang Z, Xia D, Hu Y, Geng S. Clinical Characteristics of Patients with Severe Pneumonia Caused by the SARS-CoV-2 in Wuhan, China. Respiration; international review of thoracic diseases. 2020;99:649-57.https://doi.10.1159/000507940.

[139] Wei Y, Zeng W, Huang X, Li J, Qiu X, Li H, et al. Clinical characteristics of 276 hospitalized patients with coronavirus disease 2019 in Zengdu District, Hubei Province: a single-center descriptive study. BMC infectious diseases. 2020;20:549.https://doi.10.1186/s12879-020-05252-8.

[140] Wu J, Li W, Shi X, Chen Z, Jiang B, Liu J, et al. Early antiviral treatment contributes to alleviate the severity and improve the prognosis of patients with novel coronavirus disease (COVID-19). Journal of internal medicine. 2020;288:128-38.https://doi.10.1111/joim.13063.

[141] Wu S, Du Z, Shen S, Zhang B, Yang H, Li X, et al. Identification and Validation of a Novel Clinical Signature to Predict the Prognosis in Confirmed Coronavirus Disease 2019 Patients. Clinical infectious diseases : an official publication of the Infectious Diseases Society of America. 2020;71:3154-62.https://doi.10.1093/cid/ciaa793.

[142] Xiong S, Liu L, Lin F, Shi J, Han L, Liu H, et al. Clinical characteristics of 116 hospitalized patients with COVID-19 in Wuhan, China: a single-centered, retrospective, observational study. BMC infectious diseases. 2020;20:787.https://doi.10.1186/s12879-020-05452-2.

[143] Xiong TY, Huang FY, Liu Q, Peng Y, Xu YN, Wei JF, et al. Hypertension is a risk factor for adverse outcomes in patients with coronavirus disease 2019: a cohort study. Annals of medicine. 2020;52:361-6.https://doi.10.1080/07853890.2020.1802059.

[144] Xu XW, Wu XX, Jiang XG, Xu KJ, Ying LJ, Ma CL, et al. Clinical findings in a group of patients infected with the 2019 novel coronavirus (SARS-Cov-2) outside of Wuhan, China: retrospective case series. BMJ (Clinical research ed). 2020;368:m606.https://doi.10.1136/bmj.m606.

[145] Yan X, Han X, Peng D, Fan Y, Fang Z, Long D, et al. Clinical Characteristics and Prognosis of 218 Patients With COVID-19: A Retrospective Study Based on Clinical Classification. Frontiers in medicine. 2020;7:485.https://doi.10.3389/fmed.2020.00485.

[146] Yan X, Li F, Wang X, Yan J, Zhu F, Tang S, et al. Neutrophil to lymphocyte ratio as prognostic and predictive factor in patients with coronavirus disease 2019: A retrospective cross-sectional study. Journal of medical virology. 2020;92:2573-81.https://doi.10.1002/jmv.26061.

[147] Yang Q, Xie L, Zhang W, Zhao L, Wu H, Jiang J, et al. Analysis of the clinical characteristics, drug treatments and prognoses of 136 patients with coronavirus disease 2019. Journal of clinical pharmacy and therapeutics. 2020;45:609-16.https://doi.10.1111/jcpt.13170.

[148] Yang X, Yu Y, Xu J, Shu H, Xia J, Liu H, et al. Clinical course and outcomes of critically ill patients with SARS-CoV-2 pneumonia

in Wuhan, China: a single-centered, retrospective, observational study. The Lancet Respiratory medicine. 2020;8:475-81.https://doi.10.1016/s2213-2600(20)30079-5.

[149] Yang Y, Ding L, Zou X, Shen Y, Hu D, Hu X, et al. Visceral Adiposity and High Intramuscular Fat Deposition Independently Predict Critical Illness in Patients with SARS-CoV-2. Obesity (Silver Spring, Md). 2020;28:2040-8.https://doi.10.1002/oby.22971.

[150] Yao C. Analysis of clinical and epidemiological characteristics for 92 cases of COVID-19. Prog in Microbiol Immunol. 2020:39–44

[151] Yu C, Lei Q, Li W, Wang X, Li W, Liu W. Epidemiological and clinical characteristics of 1663 hospitalized patients infected with COVID-19 in Wuhan, China: a single-center experience. Journal of infection and public health. 2020;13:1202-9.https://doi.10.1016/j.jiph.2020.07.002.

[152] Yu C, Lei Q, Li W, Wang X, Liu W, Fan X, et al. Clinical Characteristics, Associated Factors, and Predicting COVID-19 Mortality Risk: A Retrospective Study in Wuhan, China. American journal of preventive medicine. 2020;59:168-75.https://doi.10.1016/j.amepre.2020.05.002.

[153] Yuan M, Yin W, Tao Z, Tan W, Hu Y. Association of radiologic findings with mortality of patients infected with 2019 novel coronavirus in Wuhan, China. PloS one. 2020;15:e0230548.https://doi.10.1371/journal.pone.0230548.

[154] Zagidullin NS, Motloch LJ, Musin TI, Bagmanova ZA, Lakman IA, Tyurin AV, et al. J-waves in acute COVID-19: A novel disease characteristic and predictor of mortality? PloS one. 2021;16:e0257982.https://doi.10.1371/journal.pone.0257982.

[155] Zerbo O, Lewis N, Fireman B, Goddard K, Skarbinski J, Sejvar JJ, et al. Population-based assessment of risks for severe COVID-19 disease outcomes. Influenza and other respiratory viruses. 2022;16:159-65.https://doi.10.1111/irv.12901.

[156] Zhang F, Yang D, Li J, Gao P, Chen T, Cheng Z, et al. Myocardial injury is associated with in-hospital mortality of confirmed or suspected COVID-19 in Wuhan, China: A single center retrospective cohort study. medRxiv : the preprint server for health sciences. 2020:2020.03.21.20040121.https://doi.10.1101/2020.03.21.20040121.

[157] Zhang G, Hu C, Luo L, Fang F, Chen Y, Li J, et al. Clinical features and short-term outcomes of 221 patients with COVID-19 in Wuhan, China. Journal of clinical virology : the official publication of the Pan American Society for Clinical Virology. 2020;127:104364.https://doi.10.1016/j.jcv.2020.104364.

[158] Zhang H, Cao X, Kong M, Mao X, Huang L, He P, et al. Clinical and hematological characteristics of 88 patients with COVID-19. International journal of laboratory hematology. 2020;42:780-7.https://doi.10.1111/ijlh.13291.

[159] Zhang J, Liu P, Wang M, Wang J, Chen J, Yuan W, et al. The clinical data from 19 critically ill patients with coronavirus disease 2019: a single-centered, retrospective, observational study. Zeitschrift fur Gesundheitswissenschaften = Journal of public health. 2020:1-4.https://doi.10.1007/s10389-020-01291-2.

[160] Zhang JJ, Dong X, Cao YY, Yuan YD, Yang YB, Yan YQ, et al. Clinical characteristics of 140 patients infected with SARS-CoV-2 in Wuhan, China. Allergy. 2020;75:1730-41.https://doi.10.1111/all.14238.

[161] Zhang N, Zhang H, Tang Y, Zhang H, Ma A, Xu F, et al. Risk factors for illness severity in patients with COVID-19 pneumonia: a prospective cohort study. International journal of medical sciences. 2021;18:921-8.https://doi.10.7150/ijms.51205.

[162] Zhang Q, Wang Z, Lv Y, Zhao J, Dang Q, Xu D, et al. Clinical features and prognostic factors of patients with COVID-19 in Henan Province, China. Human cell. 2021;34:419-35.https://doi.10.1007/s13577-021-00499-y.

[163] Zhang Y, Xiao LS, Li P, Zhu H, Hu C, Zhang WF, et al. Clinical Characteristics of Patients With Progressive and Non-progressive Coronavirus Disease 2019: Evidence From 365 Hospitalised Patients in Honghu and Nanchang, China. Frontiers in medicine. 2020;7:556818.https://doi.10.3389/fmed.2020.556818.

[164] Zhao C, Bai Y, Wang C, Zhong Y, Lu N, Tian L, et al. Risk factors related to the severity of COVID-19 in Wuhan. International journal of medical sciences. 2021;18:120-7.https://doi.10.7150/ijms.47193.

[165] Zhao M, Wang M, Zhang J, Gu J, Zhang P, Xu Y, et al. Comparison of clinical characteristics and outcomes of patients with coronavirus disease 2019 at different ages. Aging. 2020;12:10070-86.https://doi.10.18632/aging.103298.

[166] Zhao W, Zha X, Wang N, Li D, Li A, Yu S. Clinical Characteristics and Durations of Hospitalized Patients with COVID-19 in Beijing: A Retrospective Cohort Study. Cardiovascular Innovations and Applications. 2021;6:33-

44.https://doi.10.15212/CVIA.2021.0019.

[167] Zhao XY, Xu XX, Yin HS, Hu QM, Xiong T, Tang YY, et al. Clinical characteristics of patients with 2019 coronavirus disease in a non-Wuhan area of Hubei Province, China: a retrospective study. BMC infectious diseases. 2020;20:311.https://doi.10.1186/s12879-020-05010-w.

[168] Zheng F, Tang W, Li H, Huang YX, Xie YL, Zhou ZG. Clinical characteristics of 161 cases of corona virus disease 2019 (COVID-19) in Changsha. European review for medical and pharmacological sciences. 2020;24:3404-10.https://doi.10.26355/eurrev_202003_20711.

[169] Zhou F, Yu T, Du R, Fan G, Liu Y, Liu Z, et al. Clinical course and risk factors for mortality of adult inpatients with COVID-19 in Wuhan, China: a retrospective cohort study. Lancet (London, England). 2020;395:1054-62.https://doi.10.1016/s0140-6736(20)30566-3.

[170] Zhou Y, Han T, Chen J, Hou C, Hua L, He S, et al. Clinical and Autoimmune Characteristics of Severe and Critical Cases of COVID-19. Clinical and translational science. 2020;13:1077-86.https://doi.10.1111/cts.12805.

[171] Zhou Y, He Y, Yang H, Yu H, Wang T, Chen Z, et al. Development and validation a nomogram for predicting the risk of severe COVID-19: A multi-center study in Sichuan, China. PloS one. 2020;15:e0233328.https://doi.10.1371/journal.pone.0233328.

[172] Zou W, Liu C, Cai Y, Zeng Z, Fu X. Comparison of Recovery Phase CT Features between Mild/moderate and Severe/critical Coronavirus Disease 2019 Patients. Zhongguo yi xue ke xue yuan xue bao Acta Academiae Medicinae Sinicae. 2020;42:370-5
